# An integrated method for compensating and correcting nonlinear error in five-axis machining utilizing cutter contacting point data

**DOI:** 10.1038/s41598-024-59458-w

**Published:** 2024-04-16

**Authors:** Liangji Chen, Haohao Xu, Qiang Huang, Pengcheng Wang

**Affiliations:** Key Laboratory of Advanced Manufacturing and Automation Technology (Guilin University of Technology), Education Department of Guangxi Zhuang Autonomous Region, Guilin, 541006 China

**Keywords:** CNC machining, Five-axis linear interpolation, Contour error, CC point trajectory nonlinear error, Compensating and correcting, Engineering, Mechanical engineering

## Abstract

In current five-axis computer numerical control (CNC) machining, the use of minute linear path segments as an approximation for the ideal cutter contacting (CC) point trajectory is still prevalent. However, introducing rotation axes leads to a deviation of the actual CC point trajectory from the ideal, resulting in nonlinear errors. An integrated method is proposed in this paper for compensating and correcting both the contour error, associated with the approximation of the part surface by the ideal CC point trajectory and the nonlinear error of the CC point trajectory based on the information in the CC point data. By analyzing the spatial relationship between the tool posture and the CC point path during the five-axis linear interpolation process, two adjacent machining tool positions containing CC point data information are selected as the starting and ending points of the five-axis linear interpolation machining. The ideal tool center point and the actual CC point are calculated during the interpolation process, as well as the distance and the unit vector in the perpendicular direction between the actual CC point and the ideal CC point trajectory segment. In the comprehensive error compensation and correction phase, the obtained unit vectors are used as direction vectors for error compensation, and the tool center point during interpolation is first compensated and corrected. This ensures the actual CC point and the contour curve are on the same plane. The compensation direction for contour error is calculated using the start/end tool axis vectors and the ideal CC point trajectory vectors. The size of the contour error approximating the contour curve is calculated through the chord error. A second compensation and correction are applied to the tool center point for interpolation, ultimately achieving comprehensive compensation and correction of nonlinear errors. The data calculations were conducted in the MATLAB environment using actual machining data. After compensation and correction, the contour error was reduced by 76%, the nonlinear error of the CC point trajectory decreased to below 0.88 μm, and the comprehensive nonlinear error of the CC point trajectory was reduced from 19 to 1.5 μm, a reduction of 93%. This demonstrates significant practical value in enhancing the accuracy of five-axis CNC machining. Through actual machining verification, after using the method described in this paper, the average surface roughness decreased from 1.133 to 0.220 μm, and the maximum surface roughness decreased from 6.667 to 1.240 μm. This significantly demonstrates that the compensation and correction method proposed in this paper can significantly improve the surface quality of machined parts.

## Introduction

Five-axis CNC machine tools have extensive applications in aerospace, automotive, and medical devices, primarily used for machining components with complex surfaces like impeller blades, precision molds, and ship propellers^1,2^. Five-axis CNC machines, building upon the traditional three-axis models, incorporate two additional rotation axes and can complete multiple operations in a single clamping of the workpiece. These advantages significantly enhance the machining efficiency and precision of complex surface components^3,4^. However, the current five-axis CNC machining process still utilizes numerous continuous small linear path segments to approximate the contour surface curve of the workpiece, with these tiny segments forming the trajectory of the tool-workpiece CC point during five-axis CNC machining. This traditional approximation machining approach initially results in a contour approximation error. Even if the error is not considered, it will further produce the nonlinear error of the CC point trajectory caused by the introduction of the rotation axes in the five-axis CNC interpolation process^5,6^, which in turn reduces the machining accuracy and surface machining quality of the five-axis CNC machine tool. It can be seen that effectively reducing the nonlinear error of the CC point trajectory in five-axis CNC machining to improve the accuracy of five-axis CNC machining has gradually become a key technical problem to be solved in the field of five-axis CNC machining.

In response to the issues mentioned above, researchers domestically and internationally have extensively and deeply studied the nonlinear error problem in five-axis CNC machining and proposed various implementation methods.

Wang^7^ introduced the DICT (Double Interpolation algorithm based on the Cosine Theorem) method, which uses the STE (Second-order Taylor’s Expansion) method twice within one interpolation cycle, achieving minimal feed rate fluctuations within a stable consumption time. Liu^8^ proposed a method that uses a flat-bottom cutter to substitute the cutter envelope surface with a discrete bottom circle, iterating step errors in calculations. Chen^9^ modeled the nonlinear error of the tool center point, demonstrating that, within the same interpolation cycle, the tool center point lies on the same plane, thereby converting the three-dimensional problem into a two-dimensional one for research. Wang^10^ established a motion error model of five-axis machine tools using a method based on dual quaternions and analyzed its correlation with the PIGE (Position Independent Geometric Error) definition in ISO 230-7. Zhang^11^ proposed a new pre-compensation method for contour errors in five-axis CNC machine tools. This method involves fitting the machine tool’s position commands with quintic spline functions and using analytical techniques to predict the servo system’s tracking errors, thereby pre-compensating for contour errors before machining. Li^12^ proposed a three-parameter tool path interpolation method for five-axis machining with three-dimensional tool compensation, providing a suitable method for generating three-parameter tools in five-axis parameter interpolation. Wang^13^, based on HTM (Homogeneous Transformation Matrix) theory, developed an identification model for the incremental length of the double ball screw C-axis rod, mapping the relationship between the rod length increment and C-axis geometric error parameters. On this basis, five measurement trajectories were designed to identify the geometric error of the C-axis. Zhang^14^ introduced a BP neural network model to predict step error values. Sang^15^ proposed a simplified calculation model for the deviation between the ideal cutter trajectory and the actual cutter trajectory caused by the nonlinear motion of the rotary and translational axes and an improved interpolation algorithm considering geometric deviation and motion constraints, which improves the precision of machining to a certain extent. Zhang^16^ proposed an angle single sphere domain linear interpolation method to reduce nonlinear errors. Fu^17^ compensated for geometric errors by optimizing CNC codes with particle swarm optimization algorithms. Remus^18^ introduced a method for precisely determining errors, different from the traditional chordal deviation method, describing the exact interpolation position of the tool between two contact points. Ma^19^ proposed a feasible region solution for tool orientation with nonlinear error constraints, which can effectively avoid tool interference and improve machining accuracy, yet it may concurrently impact the efficiency of interpolation. Duong^20^ proposed an OGA (Offline Gain Adjustment) method to reduce contour errors in five-axis high-speed machining. This method optimizes servo drive control gains based on model predictive control, enhancing machining accuracy by considering machine dynamics and constraints. Chen^21^ proposed an innovative analytical path-reshaping model and solution algorithm for pre-compensating contour errors in multi-axis CNC machining. This approach integrates analytical prediction of contour error, optimal path reshaping, and a decoupling solution algorithm, significantly enhancing the accuracy of five-axis machining processes.

This paper distinguishes itself from existing literature by addressing not only the reduction of contour errors but also the positional errors of the CC point due to deviations of the actual CC point trajectory from the theoretical CC point trajectory. It adopts the concept of vector composition to synthesize these errors into a comprehensive nonlinear error, which is then compensated and corrected accordingly. This innovative approach allows for a more accurate and holistic error correction mechanism in multi-axis CNC machining processes. Existing research methods mainly focus on compensating and optimizing the nonlinear error of the tool center point trajectory caused by tool swinging. However, they do not consider the actual positional error of the CC point, which ultimately leads to low control of the CC point trajectory accuracy. The reason is the lack of CC point data throughout the five-axis CNC machining process, which results in insufficient data support for correcting the position of the CC point. This deficiency hinders effective compensation and correction of the CC point positional error during CNC machining trajectory interpolation. Furthermore, the absence of CC point data information also makes it challenging to carry out effective contour error compensation during the trajectory interpolation process, thereby complicating the reduction of surface contour errors on the workpiece.

The machining path of the CC point and the associated trajectory errors are investigated in this paper. A five-axis linear interpolation method is proposed to reduce CC point trajectory errors by adjusting the tool center point’s machining trajectory. In this method, the first step involves calculating the nonlinear error and contour error of the CC point trajectory. Subsequently, the positions where these errors are maximized are identified, leading to the determination of the error compensation direction unit vector. Finally, the corrected positions of the tool center points needed to minimize both types of errors are calculated. These corrected positions are used as the output locations for interpolation to control the machine tool’s position. The subsequent content of this paper primarily includes an introduction to conventional five-axis linear interpolation methods and their error analysis, as well as the presentation of the proposed five-axis linear interpolation method and its techniques for error compensation and correction.

## Conventional five-axis linear interpolation methods

Five-axis linear interpolation is a motion control technique used in multi-axis mechanical systems. It is primarily utilized in systems requiring complex motion paths across multiple axes, such as CNC machines and robots. Five-axis linear interpolation achieves linear movement in multidimensional space by simultaneously controlling five axes (typically three linear axes and two rotation axes). Through the collaboration of mathematical algorithms and motion controllers, five-axis linear interpolation enables synchronized and precise motion control of multiple axes, allowing mechanical systems to move along specified paths. In five-axis linear interpolation, the motion trajectory is usually composed of parameters such as target positions and the speeds of each axis. By interpolating these parameters, controllers can generate corresponding motion commands, facilitating smooth movement of the mechanical system in multidimensional space.

Currently, the five-axis linear interpolation method is achieved by data sampling between the start and endpoint of the linear path segment to be interpolated. Assuming that the coordinates of the start and endpoint of the path segment in the machine tool coordinate system are $(X_{s} ,Y_{s} ,Z_{s} ,\theta_{As} ,\theta_{Cs} )$ and $(X_{e} ,Y_{e} ,Z_{e} ,\theta_{Ae} ,\theta_{Ce} )$, respectively, with a feed rate of *f*, and the CNC system interpolation cycle is *T*, then the total number of interpolation cycles required between the start and endpoint is:

1 $$ {\text{n}} = \left[ {{\text{D}}/({\text{fT}})} \right] $$

In the formula, the operator [*] represents the function of rounding * to the nearest integer, and *D* denotes the total length of the path segment to be interpolated.

2 $$ D = \sqrt {\left( {X_{e} - X_{s} } \right)^{2} + \left( {Y_{e} - Y_{s} } \right)^{2} + \left( {Z_{e} - Z_{s} } \right)^{2} } $$

Based on the principle of data sampling interpolation, it is understood that the coordinates of the interpolation points between the start and endpoint can be calculated as:

3 $$ \left\{ \begin{gathered} x_{i} = i[(X_{e} - X_{s} )/n] + X_{s} \hfill \\ y_{i} = i[(Y_{e} - Y_{s} )/n] + Y_{s} \hfill \\ z_{i} = i[(Z_{e} - Z_{s} )/n] + Z_{s} \hfill \\ \theta_{Ai} = i[(\theta_{Ae} - \theta_{As} )/n] + \theta_{As} \hfill \\ \theta_{Ci} = i[(\theta_{Ce} - \theta_{Cs} )/n] + \theta_{Cs} \hfill \\ \end{gathered} \right.(i = 1, \cdots ,n) $$

In Eq. (3), *x*_*i*_, *y*_*i*_, and *z*_*i*_ represent the target motion position coordinates of each translational axis of the machine tool for the *i*-th interpolation cycle, while ${\uptheta }_{{{\text{Ai}}}}$ and ${\uptheta }_{{{\text{Ci}}}}$, respectively, represent the target motion position coordinates for the A and C rotation axes. Through the calculation of the interpolation above point position coordinates, the five interpolation motion position coordinates are transmitted to the corresponding servo drivers, ultimately achieving the synchronous joint movement of the five feed coordinate axes of the machine tool.

## Conventional five-axis linear interpolation error analysis

While interpolating in the joint space as discussed in references^22,23^ maximizes the reduction of nonlinear errors, our decision was based on striking a balance between computational complexity and the precision requirements of the specific machining process we studied. Interpolation in task space, although advantageous in certain scenarios, could introduce its own challenges in terms of computational demands and the complexity of implementing real-time control systems for high-speed machining.

### Tool center point trajectory error

**Figure 1:**
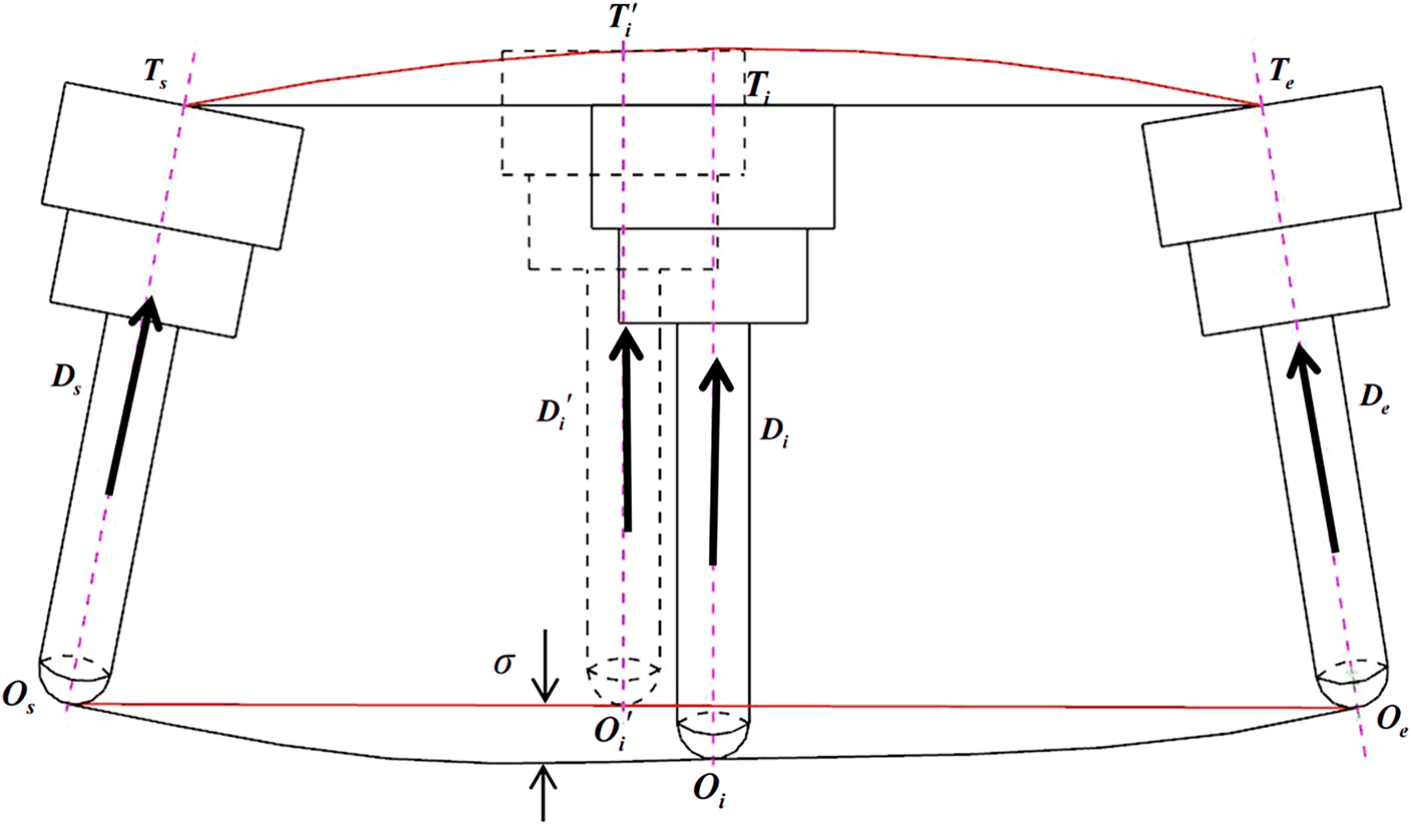
Generation of nonlinear error of five-axis linear interpolation.

Through the interpolation calculation process of the aforementioned conventional five-axis linear interpolation, it is known that after the combined movement of translational and rotation axes, the resulting motion trajectory at the tool will be as shown in Fig. 1. *O*_*s*_ and *O*_*e*_ represent the tool center point position vectors in the workpiece coordinate system corresponding to the start point $\left( {X_{s} ,Y_{s} ,Z_{s} ,\theta_{As} ,\theta_{Cs} } \right)$ and end point $\left( {X_{e} ,Y_{e} ,Z_{e} ,\theta_{Ae} ,\theta_{Ce} } \right)$ of the path segment to be interpolated. *D*_*s*_ and *D*_*e,*_ respectively, represent the unit vectors in the direction of the tool axis at the start and endpoint. In an ideal scenario of five-axis CNC machining, the tool follows the red path depicted in Fig. 1 for cutting operations, which can produce a workpiece with a high-quality contour surface.

However, due to the interconnected relationship between translational and rotation axes during the actual cutting motion, the tool performs a rotation movement around its pivot point while simultaneously translating. This leads to the tool center point actually following the black path depicted in Fig. 1 for the cutting motion. Consequently, during the interpolation process, a positional deviation $\sigma$ occurs between the actual interpolated cutter location $\left( {{\text{O}}_{{\text{i}}} {\text{,D}}_{{\text{i}}} } \right)$ at the *i*-th point and the anticipated ideal interpolated cutter location. This deviation represents the nonlinear error in the tool center point trajectory during five-axis linear interpolation.

To calculate the tool center point trajectory nonlinear error generated during conventional five-axis linear interpolation, one can first determine the tool axis point position vectors corresponding to Fig. 1, based on the tool center point position vectors at the start and endpoints.

4 $$ \left\{ \begin{gathered} {\mathbf{T}}_{s} {\mathbf{ = O}}_{s} {\mathbf{ + }}{\text{L}}{\mathbf{D}}_{s} \hfill \\ {\mathbf{T}}_{e} {\mathbf{ = O}}_{e} {\mathbf{ + }}{\text{L}}{\mathbf{D}}_{e} \hfill \\ \end{gathered} \right. $$

In the above formula, *L* represents the total length from the tool center point to the tool rotation pivot point (swing tool length). Referring to Eq. (3), the position coordinates of the tool axis point corresponding to the *i*-th interpolated tool center point can be calculated as:

5 $$ {\mathbf{T}}_{i} {\mathbf{ = T}}_{s} {\mathbf{ + (T}}_{e} {\mathbf{ - T}}_{s} {\mathbf{)*}}{\text{i}}/n $$

Utilizing the two rotation motion coordinates in Eq. (3), the unit vector of the cutter axis at the *i*-th interpolated tool center point can be derived as follows:

6 $$ \left\{ \begin{gathered} I_{i} = \sin \theta_{Ai} \sin \theta_{Ci} \hfill \\ J_{i} = - \sin \theta_{Ai} \cos \theta_{Ci} \hfill \\ K_{i} = \cos \theta_{Ai} \hfill \\ \end{gathered} \right. $$

Combining Eq. (4), the expression for the position vector ***O***_*i*_ of the *i*-th interpolated tool center point can be calculated as

7 $$ \left\{ \begin{gathered} X_{i} = (X_{s} + LI_{s} ) + i[(X_{e} + LI_{e} - X_{s} - LI_{s} )/n] - LI_{i} \hfill \\ Y_{i} = (Y_{s} + LJ_{s} ) + i[(Y_{e} + LJ_{e} - Y_{s} - LJ_{s} )/n] - LJ_{i} \hfill \\ Z_{i} = (Z_{s} + LK_{s} ) + i[(Z_{e} + LK_{e} - Z_{s} - LK_{s} )/n] - LK_{i} \hfill \\ \end{gathered} \right. $$

Therefore, the nonlinear error $\sigma_{i}$ at the *i*-th interpolated tool center point is the distance from point ***O***_*i*_ to the spatial line ***O***_*s*_***O***_*e*_, which can be calculated as

8 $$ \sigma_{i} = \frac{{\left| {{\mathbf{a}} \times {\mathbf{b}}} \right|}}{{\left| {\mathbf{a}} \right|}} $$

In Eq. (8), ***a*** = ***O***_*e*_ − ***O***_*s*_, ***b*** = ***O***_*i*_ − ***O***_*s*_.

### CC point trajectory error

Having discussed the errors associated with the tool center point, it is essential to now shift the focus to the trajectory errors of the CC point. Understanding the distinction between the tool center point and the CC point is crucial for a comprehensive grasp of machining accuracy. While the tool center point errors illuminate the spatial positioning of the tool itself, the CC point errors delve into the specifics of how the tool interacts with the material being machined. This aspect is vital for assessing the quality of the machined surface and the efficiency of material removal. The following discussion will explore the nuances of CC point trajectory errors, highlighting how these differ from and impact the errors associated with the tool center point. This examination is pivotal for optimizing machining processes and enhancing overall workpiece quality.

#### CC point trajectory chord error

**Figure 2:**
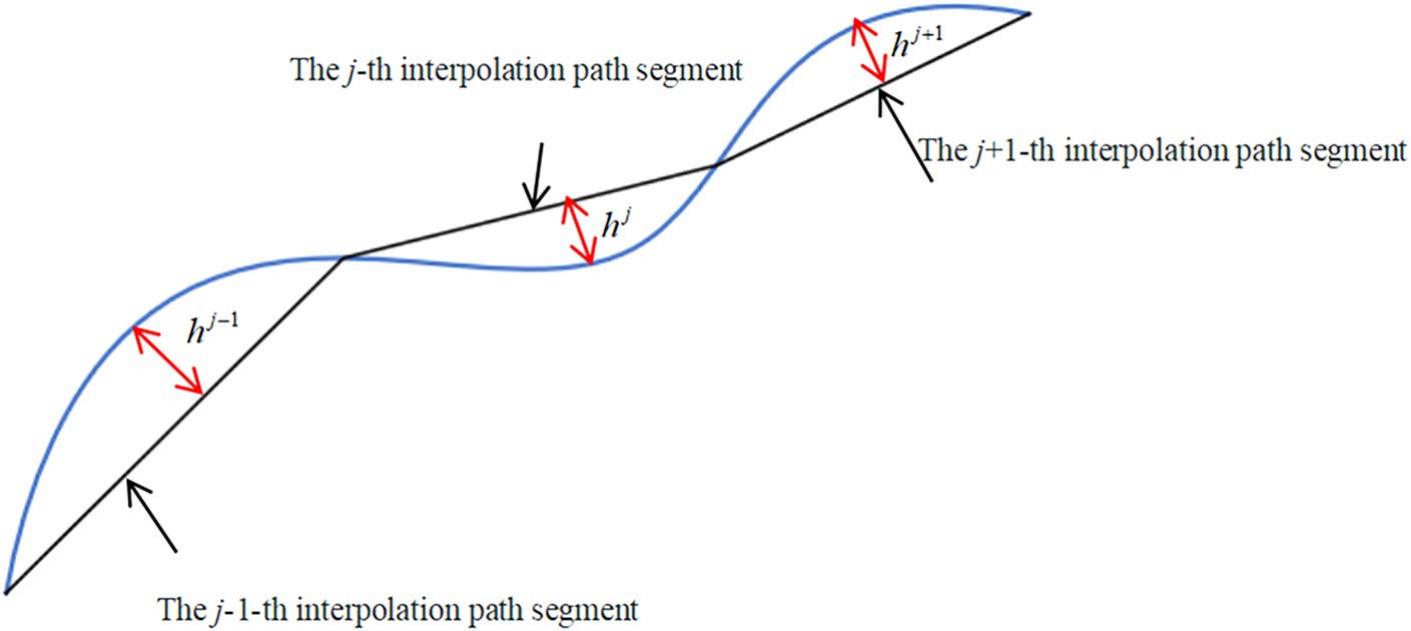
Chord error in CC point trajectory.

In current five-axis machining CNC programming, the common practice involves extracting several discrete data points within the permissible accuracy range on the surface contour of the workpiece to serve as CC points. These points are then connected by line segments, forming the ideal trajectory of the CC points. When this continuous ideal CC point trajectory is used as an interpolation path segment to approximate a designed curve on the workpiece contour surface (contour design curve), each interpolation path segment (the *j*-th segment in Fig. 2) will generate a distance error (*h*^*j*^ in Fig. 2) relative to the contour design curve. This error, inherent in CNC programming, is defined as the CC point trajectory chord error.

**Figure 3:**
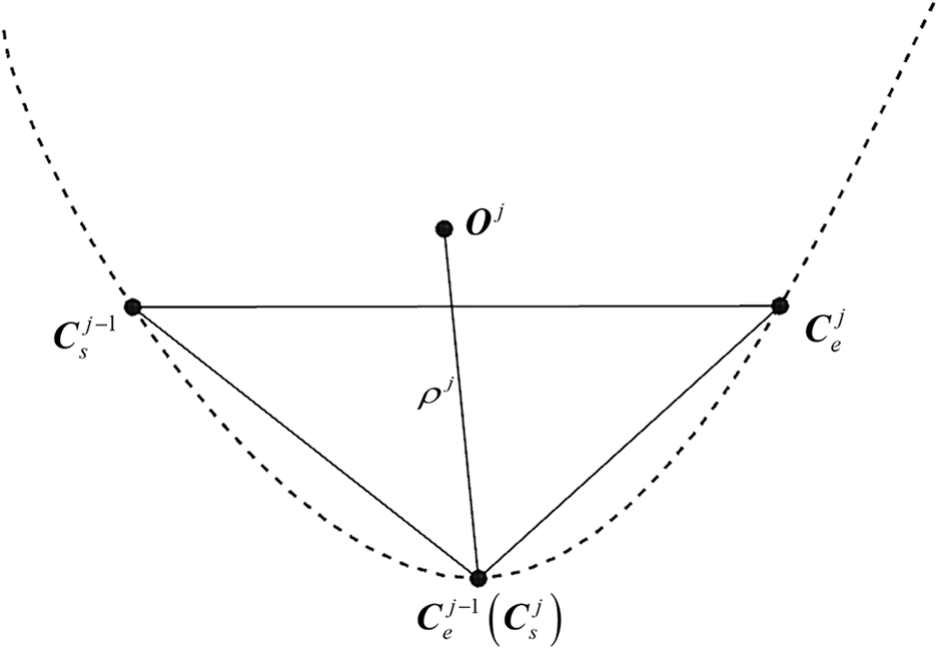
The curvature circle and curvature radius at the CC point.

Due to the complexity of accurately calculating the chord error in the CC point trajectory, an arc model is typically used to approximate the solution of the tool path. To calculate the chord error of the CC point trajectory, it is first necessary to determine the curvature radius of the contour design curve based on the approximate circular arc formed by three adjacent CC points. As shown in Fig. 3, ${\varvec{C}}_{s}^{j - 1}$ and ${\varvec{C}}_{e}^{j - 1}$ represent the starting and ending CC points of the *j*-1th interpolation path segment, while ${\varvec{C}}_{s}^{j}$ and ${\varvec{C}}_{e}^{j}$ denote the starting and ending CC points of the* j*-th interpolation path segment.

The curvature radius ${\uprho }^{j}$ at the starting CC point of the *j*-th interpolation path segment can be calculated as

9 $$ \rho^{j} = \frac{{|{\mathbf{C}}_{e}^{j} - {\mathbf{C}}_{s}^{j} | \cdot |{\mathbf{C}}_{s}^{{j - {1}}} - {\mathbf{C}}_{e}^{{j - {1}}} | \cdot |{\mathbf{C}}_{e}^{j} - {\mathbf{C}}_{s}^{{j - {1}}} |}}{{2|({\mathbf{C}}_{e}^{j} - {\mathbf{C}}_{s}^{j} ) \times ({\mathbf{C}}_{e}^{{j - {1}}} - {\mathbf{C}}_{s}^{{j - {1}}} )|}} $$

**Figure 4:**
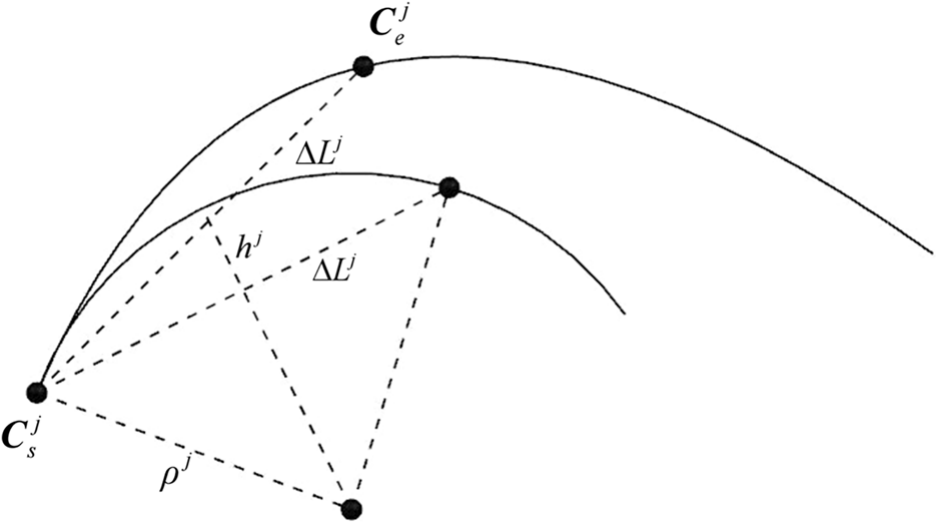
Estimating chord error using the approximate circular arc method.

The chord error at this CC point can be obtained using the circular arc approximation method, as illustrated in Fig. 4.

10 $$ h^{j} = \rho^{j} - \sqrt {(\rho^{j} )^{2} - (\Delta L^{j} /2)^{2} } $$

The equation,$\Delta {\text{L}}^{j}$ represents the distance between the given CC point and its adjacent CC point, which can be calculated using the following formula:

11 $$ \Delta L^{j} = {\mathbf{|C}}_{e}^{j} {\mathbf{ - C}}_{s}^{j} {\mathbf{|}} $$

#### CC point trajectory nonlinear error

**Figure 5:**
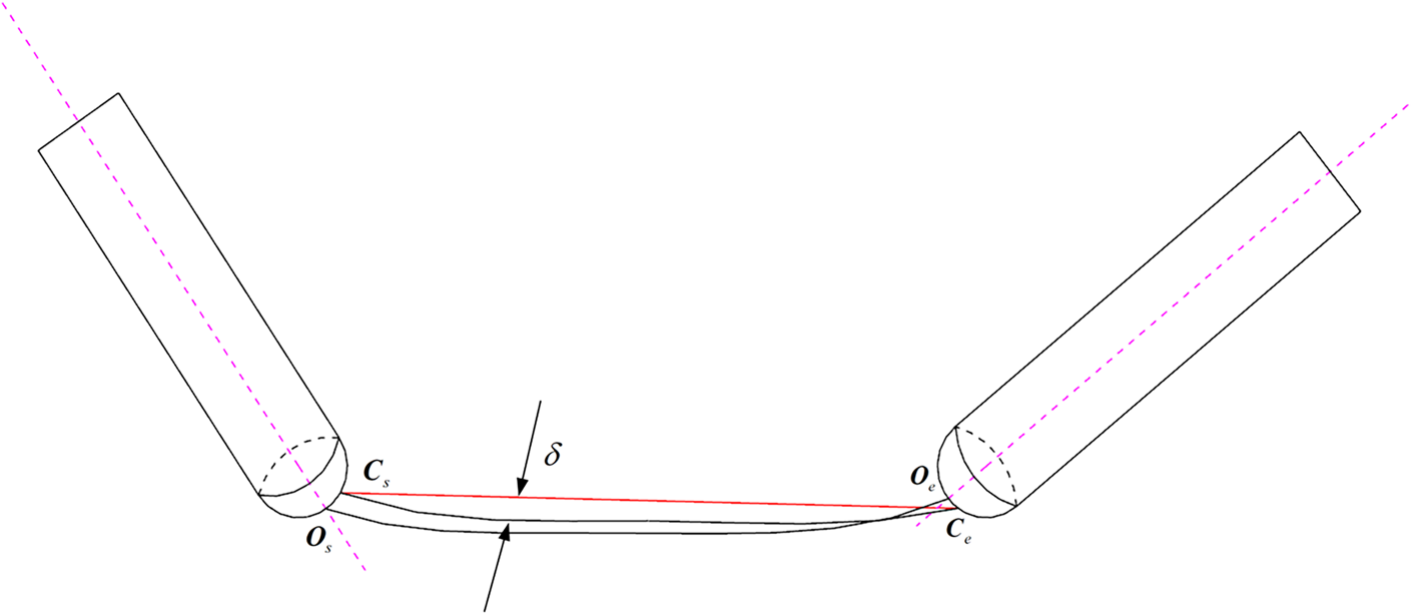
Nonlinear error in CC point trajectory.

Unlike chord error, which is caused by approximating the tool path with tiny linear segments instead of a smooth, continuous trajectory, nonlinear error arises from the tool’s oscillation or deviation from the predetermined path. During the conventional five-axis linear interpolation process, the combined movement of three translational axes and two rotation axes can cause the actual CC point’s motion trajectory to deviate from the ideal CC point trajectory, resulting in nonlinear errors in the CC point trajectory. This deviation arises because, following the linear interpolation movement through the CNC system, only the precision of the CC points at the start and endpoint can be guaranteed. It cannot ensure that the CC points, after tool rotation at intermediate interpolation points, still remain on the ideal CC point trajectory. Thus, the actual CC point trajectory between ***C***_*s*_ and ***C***_*e*_ (the black curve in Fig. 5) deviates from the ideal CC point trajectory (the red line segment ***C***_*s*_***C***_*e*_ in Fig. 5). The distance *δ* between the two trajectories represents the nonlinear error of the CC point trajectory.

## Five-axis linear interpolation method incorporating CC points

The conventional five-axis linear interpolation methods exhibit errors in both tool center point and CC point trajectories. Current technological approaches, due to the lack of relevant data on the CC points, are unable to effectively reduce these trajectory errors. Therefore, it is necessary to propose a new method of five-axis linear interpolation, distinct from conventional approaches, that allows comprehensive compensation and correction of both types of trajectory errors during the interpolation process. To ensure that the compensation and correction of trajectory errors are data-supported, a new five-axis linear interpolation method that incorporates CC point data is introduced in this paper.

From the cutter location file, two adjacent cutter location data are extracted. Let’s assume that the cutter location data for the cutting start and end points are respectively $\left( {{\varvec{X}}_{{\varvec{s}}} {,}{\varvec{Y}}_{{\varvec{s}}} {,}{\varvec{Z}}_{{\varvec{s}}} {,}{\varvec{I}}_{{\varvec{s}}} {,}{\varvec{J}}_{{\varvec{s}}} {,}{\varvec{K}}_{{\varvec{s}}} {,}\user2{X^{\prime}}_{{\varvec{s}}} {,}\user2{Y^{\prime}}_{{\varvec{s}}} {,}\user2{Z^{\prime}}_{{\text{s}}} } \right)$ and $\left( {{\varvec{X}}_{e} {,}{\varvec{Y}}_{e} {,}{\varvec{Z}}_{e} {,}{\varvec{I}}_{e} {,}{\varvec{J}}_{e} {,}{\varvec{K}}_{e} {,}\user2{X^{\prime}}_{e} {,}\user2{Y^{\prime}}_{e} {,}\user2{Z^{\prime}}_{e} } \right)$, where (*X, Y, Z*) represents the coordinates of the tool center point, (*I, J, K*) the unit vector of the cutter axis, and (*X′, Y′, Z′*) the coordinates of the CC point.

Based on the structure of the five-axis CNC machine tool being used (taking an A-C type dual-rotary table CNC machine as an example), the angular displacements of the two rotation axes can always be calculated from the unit vector of the cutter axis.

12 $$ \left\{ \begin{gathered} \theta_{A} = arc\cos K \hfill \\ \theta_{C} = \left\{ \begin{gathered} \pi /2 - \arctan (J/I) \, (I > 0,J < 0) \hfill \\ \pi /2 + \arctan (J/I){ (}I > 0,J > 0) \hfill \\ 3\pi /2 - \arctan (J/I) \, (I < 0,J < 0) \hfill \\ 3\pi /2 + \arctan (J/I) \, (I < 0,J > 0) \hfill \\ \end{gathered} \right. \hfill \\ \end{gathered} \right. $$

Utilizing Eq. (3), the rotation motion coordinates for the *i*-th interpolation cycle can be calculated. By employing Eqs. (4), (5), (6), and (7), the position coordinates of the tool center point for the *i*-th interpolation cycle can be determined, as well as the calculation of the corresponding ideal CC point position coordinates for the *i*-th interpolation cycle.

13 $$ \left\{ \begin{gathered} X^{\prime}_{i} = X^{\prime}_{s} + i/n * (X^{\prime}_{e} - X^{\prime}_{s} ) \hfill \\ Y^{\prime}_{i} = Y^{\prime}_{s} + i/n * (Y^{\prime}_{e} - Y^{\prime}_{s} ) \hfill \\ Z^{\prime}_{i} = Z^{\prime}_{s} + i/n * (Z^{\prime}_{e} - Z^{\prime}_{s} ) \hfill \\ \end{gathered} \right. $$

## Interpolation error compensation and correction based on CC point information

As can be understood from Sect. "Conventional five-axis linear interpolation methods", the calculation of interpolation errors in conventional five-axis linear interpolation methods includes CC point data information. However, due to the lack of detailed descriptions of the CC point position data in the interpolation data, it is not feasible to calculate the position error of the interpolated CC point and the contour error, let alone carry out error compensation and correction during the interpolation process. This section will detail the derivation of the calculation of errors in the interpolation of CC points separately. Based on the calculation results, compensation and correction for the errors obtained in the workpiece coordinate system will be conducted. This ensures that errors are assessed based on the final expected geometric shape of the workpiece, thereby directly demonstrating machining accuracy from the perspective of the finished product.

### Calculation of interpolated CC point position error

**Figure 6:**
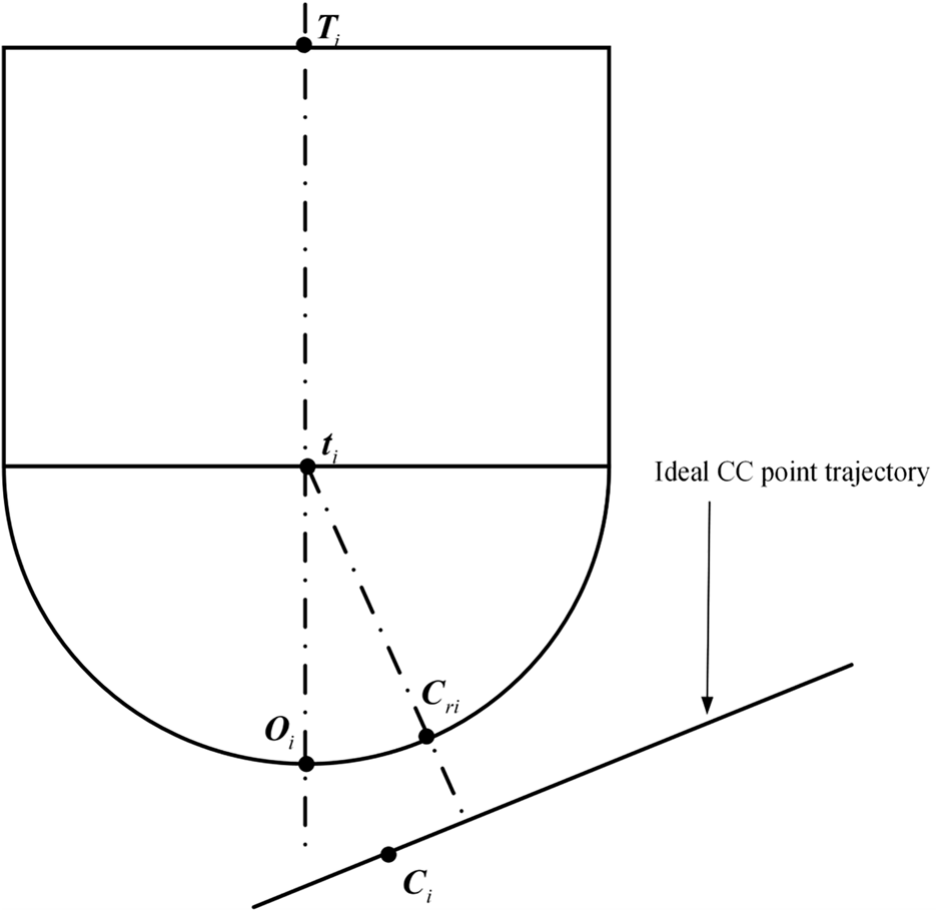
The spatial relationship between the tool and the ideal CC point trajectory.

Based on Eqs. (5), (6), and (7), the tool axis point vector ***T***_*i*_, cutter axis unit vector ***D***_*i*_, and tool center point vector ***O***_*i*_ for the *i*-th interpolation cycle can be calculated, respectively. Utilizing the coordinate vectors ***O***_*i*_, ***T***_*i*_, and ***C***_*i*_, the spatial relationship between the tool and the ideal CC point trajectory during the *i*-th interpolation cycle can be depicted, as shown in Fig. 6. This research concentrates on the application of ball end cutters in multi-axis CNC machining processes and their impact on machining accuracy. Due to their unique geometric shape, ball end cutters necessitate a different method for calculating the actual CC point compared to flat end cutters, which in turn affects the implementation of error compensation strategies.

**Figure 7:**
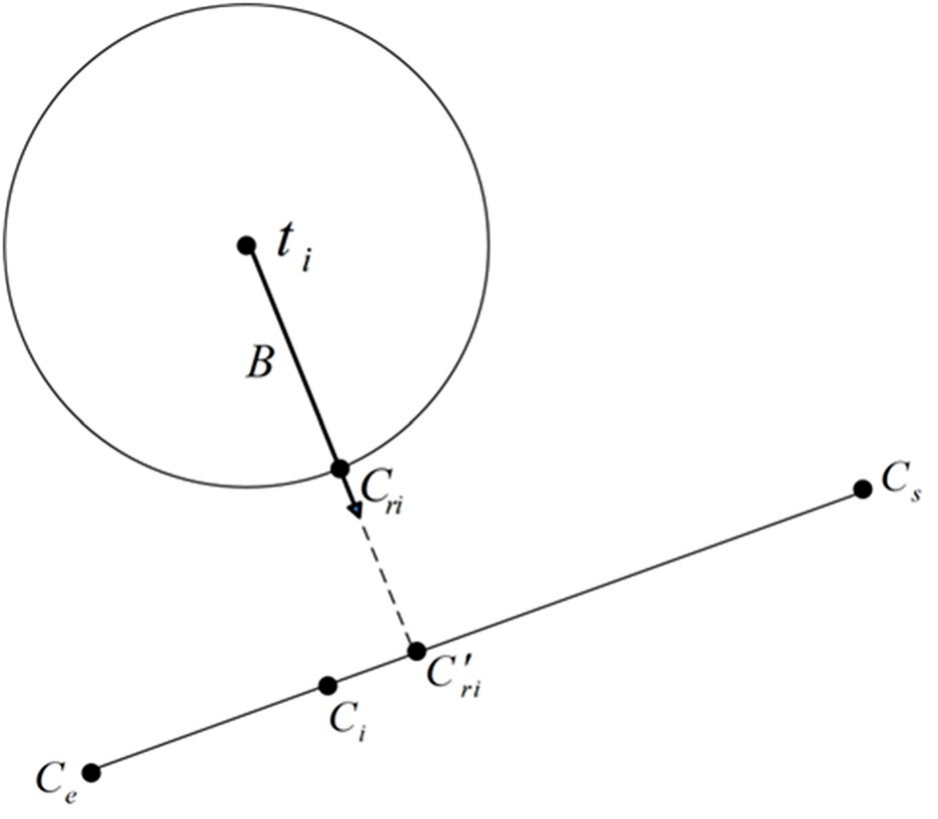
Local magnification during full path segment interpolation.

As can be seen from Fig. 6, the position coordinates of the ball center point can be expressed as

14 $$ {\mathbf{t}}_{i} {\mathbf{ = O}}_{i} + r{\mathbf{D}}_{i} $$

where *r* is the tool radius. Magnifying a section of Fig. 6 results in the enlarged view of the entire path segment interpolation as shown in Fig. 7.

A perpendicular line ${\mathbf{t}}_{i} {\mathbf{C^{\prime}}}_{ri}$ is drawn from the ball center point ***t***_*i*_ to the line segment ***C***_*s*_***C***_*e*_, with the foot of perpendicular being the point ${\mathbf{C^{\prime}}}_{{{\mathbf{ri}}}}$. The perpendicular line ${\mathbf{t}}_{{\mathbf{i}}} {\mathbf{C^{\prime}}}_{{{\mathbf{ri}}}}$ intersects the cutter edge contour line at ***C***_*ri*_. The intersection point ***C***_*ri*_ represents the nearest point to the ideal trajectory of the CC point ***C***_*s*_***C***_*e*_, and it is considered the actual CC point. The unit vector ***B*** in the direction of nonlinear error compensation and correction can be calculated as

15 $$ {\mathbf{B}} = \frac{{{\mathbf{C^{\prime}}}_{{{\mathbf{ri}}}} {\mathbf{ - t}}_{i} }}{{\left| {{\mathbf{C^{\prime}}}_{{{\mathbf{ri}}}} {\mathbf{ - t}}_{i} } \right|}} = \frac{{\left[ {{\mathbf{d}} \times {\mathbf{e}}} \right] \times {\mathbf{e}}}}{{\left| {\left[ {{\mathbf{d}} \times {\mathbf{e}}} \right] \times {\mathbf{e}}} \right|}} $$

In the above equation, ${\mathbf{d}} = {\mathbf{t}}_{i} - {\mathbf{C}}_{{\mathbf{s}}}$, ${\mathbf{e}} = {\mathbf{C}}_{e} - {\mathbf{C}}_{s}$.

Assuming the foot of the perpendicular ${\mathbf{C^{\prime}}}_{{{\mathbf{ri}}}} = (X^{\prime}_{c} ,Y^{\prime}_{c} ,Z^{\prime}_{c} )$, based on the equation of the linear path segment ***C***_*s*_***C***_*e*_ of the CC point

16 $$ \frac{{X^{\prime}_{c} - X^{\prime}_{s} }}{{X^{\prime}_{e} - X^{\prime}_{s} }} = \frac{{Y^{\prime}_{c} - Y^{\prime}_{s} }}{{Y^{\prime}_{e} - Y^{\prime}_{s} }} = \frac{{Z^{\prime}_{c} - Z^{\prime}_{s} }}{{Z^{\prime}_{e} - Z^{\prime}_{s} }} $$

The equation of the plane passing through a point ${\mathbf{t}}_{i} = (X^{\prime}_{o} ,Y^{\prime}_{o} ,Z^{\prime}_{o} )$ and perpendicular to the line ***C***_*s*_***C***_*e*_ can be derived as

17 $$ (X^{\prime}_{e} - X^{\prime}_{s} )(X^{\prime}_{c} - X^{\prime}_{o} ) + (Y^{\prime}_{e} - Y^{\prime}_{s} )(Y^{\prime}_{c} - Y^{\prime}_{o} ) + (Z^{\prime}_{e} - Z^{\prime}_{s} )(Z^{\prime}_{c} - Z^{\prime}_{o} ) = 0 $$

By combining Eqs. (16) and (17), the foot of perpendicular ${\mathbf{C^{\prime}}}_{{{\mathbf{ri}}}}$ can be calculated.

Using Eq. (18), the actual CC point position vector for the *i*-th interpolation cycle can be calculated.

18 $$ {\mathbf{C}}_{ri} = {\mathbf{t}}_{i} + r{\mathbf{B}} $$

Therefore, the position error at the interpolated CC point can be calculated as

19 $$ {\updelta }_{{\text{i}}} = \left| {{\mathbf{C^{\prime}}}_{ri} - {\mathbf{t}}_{i} } \right| - {\text{r}} = \frac{{\left| {{\mathbf{e}} \times {\mathbf{d}}} \right|}}{{\left| {\mathbf{e}} \right|}} - {\text{r}} $$

### Approximate calculation of interpolated CC point contour error

**Figure 8:**
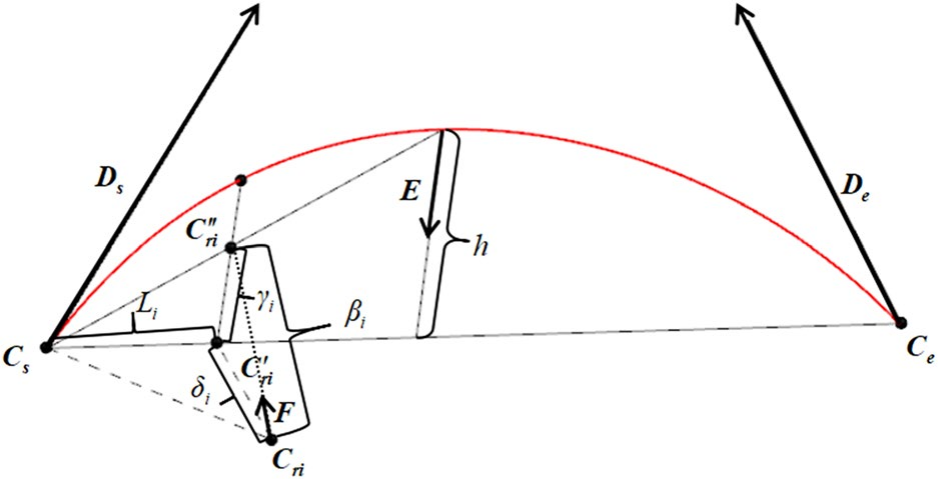
Approximate calculation of contour error.

For the contour design curve represented by the red line in Fig. 8, an approximate calculation process for the contour error at the CC point within any interpolation cycle can be performed as follows:

The chord error *h* at the midpoint of the segment ***C***_*s*_***C***_*e*_ is approximated as the maximum error. A perpendicular line ${\mathbf{C^{\prime}}}_{{{\mathbf{ri}}}} {\mathbf{C^{\prime\prime}}}_{{{\mathbf{ri}}}}$ is drawn from the point ${\mathbf{C^{\prime}}}_{{{\mathbf{ri}}}}$ to the segment ***C***_*s*_***C***_*e*_, intersecting the contour design curve at the point ${\mathbf{C^{\prime\prime}}}_{{{\mathbf{ri}}}}$. The unit vector ***E*** directed from ${\mathbf{C^{\prime\prime}}}_{{{\mathbf{ri}}}}$ towards ${\mathbf{C^{\prime}}}_{{{\mathbf{ri}}}}$ is calculated as:

20 $$ {\mathbf{E}} = \frac{{{\mathbf{e}} \times \left[ {{\mathbf{D}}_{s} \times {\mathbf{D}}_{e} } \right]}}{{\left| {{\mathbf{e}} \times \left[ {{\mathbf{D}}_{s} \times {\mathbf{D}}_{e} } \right]} \right|}} $$

In Eq. (20), ***D***_*s*_ and ***D***_*e****,***_ respectively, represent the unit vectors of the cutter axis at the start and endpoints.

The length of the segment ${\mathbf{C}}_{{\mathbf{s}}} {\mathbf{C^{\prime}}}_{{{\mathbf{ri}}}}$ can be calculated as

21 $$ L_{i} = \sqrt {\left| {{\mathbf{C}}_{ri} - {\mathbf{C}}_{s} } \right|^{2} - \left| {{\mathbf{C}}_{ri} - {\mathbf{C^{\prime}}}_{ri} } \right|^{2} } $$

In Eq. (21),$$\left| {{\mathbf{C}}_{{{\mathbf{ri}}}} - {\mathbf{C^{\prime}}}_{{{\mathbf{ri}}}} } \right| = \delta_{i}$$.

From the proportional relationship between the sides of similar triangles, it is known that

22 $$ \left\{ \begin{gathered} \frac{{L_{i} }}{{|{\mathbf{C}}_{e} - {\mathbf{C}}_{s} |/2}} = \frac{{\gamma_{i} }}{h} \, (L_{i} \le |{\mathbf{C}}_{e} - {\mathbf{C}}_{s} |/2) \hfill \\ \frac{{|{\mathbf{C}}_{e} - {\mathbf{C}}_{s} | - L_{i} }}{{|{\mathbf{C}}_{e} - {\mathbf{C}}_{s} |/2}} = \frac{{\gamma_{i} }}{h} \, (L_{i} > |{\mathbf{C}}_{e} - {\mathbf{C}}_{s} |/2) \hfill \\ \end{gathered} \right. $$

In Eq. (22), *h* represents the chord error of the interpolation path segment, which can be obtained by calculating using Eq. (10). Further, the contour error *γ*_*i*_ at the *i*-th interpolated CC point can be calculated as:

23 $$ \left\{ \begin{gathered} \gamma_{i} = \frac{{2L_{i} }}{{|{\mathbf{C}}_{e} - {\mathbf{C}}_{s} |}} \times h \, ({\text{L}}_{{\text{i}}} \le |{\mathbf{C}}_{e} - {\mathbf{C}}_{s} |/2) \hfill \\ \gamma_{i} = \frac{{2(|{\mathbf{C}}_{{\mathbf{e}}} - {\mathbf{C}}_{{\mathbf{s}}} | - L_{i} )}}{{|{\mathbf{C}}_{e} - {\mathbf{C}}_{s} |}} \times h \, ({\text{L}}_{{\text{i}}} > |{\mathbf{C}}_{e} - {\mathbf{C}}_{s} |/2) \hfill \\ \end{gathered} \right. $$

### Comprehensive compensation and correction of interpolation errors

In Fig. 8, *β*_*i*_ represents the combined nonlinear interpolation error formed by the contour error and the position error of the CC point at the *i*-th interpolated point, which can be calculated using the following formula.

24 $$ {\upbeta }_{{\text{i}}} = \left| {{\mathbf{C}}_{ri} {\mathbf{C^{\prime\prime}}}_{ri} } \right| = \left| {{\mathbf{C}}_{ri} {\mathbf{C^{\prime}}}_{ri} + {\mathbf{C^{\prime}}}_{ri} {\mathbf{C^{\prime\prime}}}_{ri} } \right| = \left| {{\updelta }_{{\text{i}}} {\mathbf{B}} - {\upgamma }_{{\text{i}}} {\mathbf{E}}} \right| $$

By substituting Eqs. (15), (19), (20), and (23) into Eq. (24) and rearranging, the following can be obtained.

25 $$ {\upbeta }_{{\text{i}}} = \left| {\left( {\frac{{|{\mathbf{e}} \times {\mathbf{d}}|}}{{|{\mathbf{e}}|}} - {\text{r}}} \right) \cdot \frac{{\left[ {{\mathbf{d}} \times {\mathbf{e}}} \right] \times {\mathbf{e}}}}{{|\left[ {{\mathbf{d}} \times {\mathbf{e}}} \right] \times {\mathbf{e}}|}} - \frac{k \times h}{{|{\mathbf{C}}_{{\mathbf{e}}} - {\mathbf{C}}_{{\mathbf{s}}} |}} \cdot \frac{{{\mathbf{e}} \times [{\mathbf{D}}_{s} \times {\mathbf{D}}_{e} ]}}{{|{\mathbf{e}} \times [{\mathbf{D}}_{s} \times {\mathbf{D}}_{e} ]|}}} \right| $$

In Eq. (25)

$$ k = \left\{ \begin{gathered} 2L_{i} \, ({\text{L}}_{{\text{i}}} \le |{\mathbf{C}}_{e} - {\mathbf{C}}_{s} |/2) \hfill \\ 2(|{\mathbf{C}}_{e} - {\mathbf{C}}_{s} | - L_{i} ) \, ({\text{L}}_{{\text{i}}} > |{\mathbf{C}}_{e} - {\mathbf{C}}_{s} |/2) \hfill \\ \end{gathered} \right. $$

The unit vector ***F*** for the direction of compensation and correction of the comprehensive interpolation error is:

26 $$ {\mathbf{F}} = \frac{{{\mathbf{B}} - {\mathbf{E}}}}{{\left| {{\mathbf{B}} - {\mathbf{E}}} \right|}} = \frac{{\frac{{\left[ {{\mathbf{d}} \times {\mathbf{e}}} \right] \times {\mathbf{e}}}}{{|\left[ {{\mathbf{d}} \times {\mathbf{e}}} \right] \times {\mathbf{e}}|}} - \frac{{{\mathbf{e}} \times [{\mathbf{D}}_{s} \times {\mathbf{D}}_{e} ]}}{{|{\mathbf{e}} \times [{\mathbf{D}}_{s} \times {\mathbf{D}}_{e} ]|}}}}{{\left| {\frac{{\left[ {{\mathbf{d}} \times {\mathbf{e}}} \right] \times {\mathbf{e}}}}{{|\left[ {{\mathbf{d}} \times {\mathbf{e}}} \right] \times {\mathbf{e}}|}} - \frac{{{\mathbf{e}} \times [{\mathbf{D}}_{s} \times {\mathbf{D}}_{e} ]}}{{|{\mathbf{e}} \times [{\mathbf{D}}_{s} \times {\mathbf{D}}_{e} ]|}}} \right|}} $$

Based on Eqs. (25) and (26), the coordinates of the tool center point of the new interpolated point after compensation can be calculated.

27 $$ {\mathbf{O^{\prime}}}_{i} = {\mathbf{O}}_{i} + \beta_{i} {\mathbf{F}} $$

By substituting Eqs. (24) and (26) into Eq. (27), the following can be obtained:

28 $$ \user2{O^{\prime}}_{i} = {\mathbf{O}}_{i} + \left| {(\frac{{|{\mathbf{e}} \times {\mathbf{d}}|}}{{|{\mathbf{e}}|}} - r) \cdot \frac{{\left[ {{\mathbf{d}} \times {\mathbf{e}}} \right] \times {\mathbf{e}}}}{{|\left[ {{\mathbf{d}} \times {\mathbf{e}}} \right] \times {\mathbf{e}}|}} - \frac{k \times h}{{|{\mathbf{C}}_{{\mathbf{e}}} - {\mathbf{C}}_{{\mathbf{s}}} |}} \cdot \frac{{{\mathbf{e}} \times [{\mathbf{D}}_{s} \times {\mathbf{D}}_{e} ]}}{{|{\mathbf{e}} \times [{\mathbf{D}}_{s} \times {\mathbf{D}}_{e} ]|}}} \right| \cdot \frac{{\frac{{\left[ {{\mathbf{d}} \times {\mathbf{e}}} \right] \times {\mathbf{e}}}}{{|\left[ {{\mathbf{d}} \times {\mathbf{e}}} \right] \times {\mathbf{e}}|}} - \frac{{{\mathbf{e}} \times [{\mathbf{D}}_{s} \times {\mathbf{D}}_{e} ]}}{{|{\mathbf{e}} \times [{\mathbf{D}}_{s} \times {\mathbf{D}}_{e} ]|}}}}{{\left| {\frac{{\left[ {{\mathbf{d}} \times {\mathbf{e}}} \right] \times {\mathbf{e}}}}{{|\left[ {{\mathbf{d}} \times {\mathbf{e}}} \right] \times {\mathbf{e}}|}} - \frac{{{\mathbf{e}} \times [{\mathbf{D}}_{s} \times {\mathbf{D}}_{e} ]}}{{|{\mathbf{e}} \times [{\mathbf{D}}_{s} \times {\mathbf{D}}_{e} ]|}}} \right|}} $$

**Figure 9:**
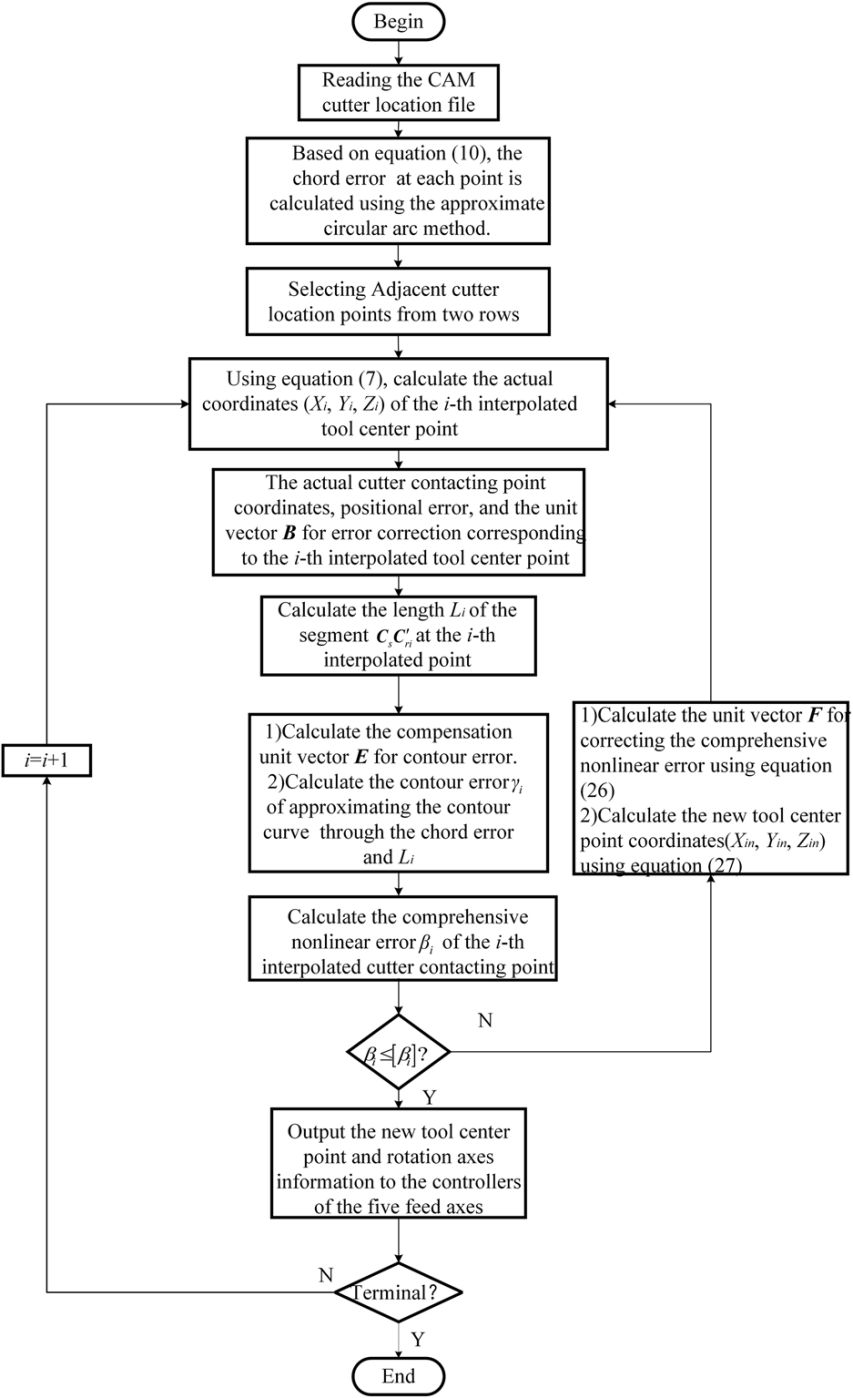
Comprehensive compensation and correction process for interpolation error.

The calculation process for the comprehensive compensation and correction of interpolation nonlinear errors is now summarized and illustrated in Fig. 9.

Using C++ to read the cutter location file generated by UG (Unigraphics), the nine coordinates of discrete data are extracted. The curvature radius at each point is calculated using Eq. (9), and then the chord error at each point is determined using the approximate circular arc method. By selecting adjacent cutter location data, the actual coordinates of the tool center point are calculated, followed by determining the actual CC point coordinates. Using Eqs. (15) and (19), the magnitude of the positional error at the CC point and the unit vector for error correction are calculated sequentially. Then, the compensation direction of the contour error is determined using the start/end tool axis vectors and the ideal CC point trajectory vectors. The magnitude of the contour error approximating the contour curve is calculated through chord error, and unit vectors for correcting both types of errors are assigned their respective magnitudes for vectors synthesis. Using Eqs. (24) and (26) are used in sequence to calculate the magnitude of the combined nonlinear error at the CC point and the unit vector for the error correction position. A comprehensive correction is then applied to the tool center point to obtain new tool center point coordinates. These new coordinates, along with the rotation axes information, are transmitted to the controllers of the five feed axes. The process continues in a loop until the endpoint is reached, checking at each iteration whether the endpoint has been achieved.

## Computational example

**Figure 10:**
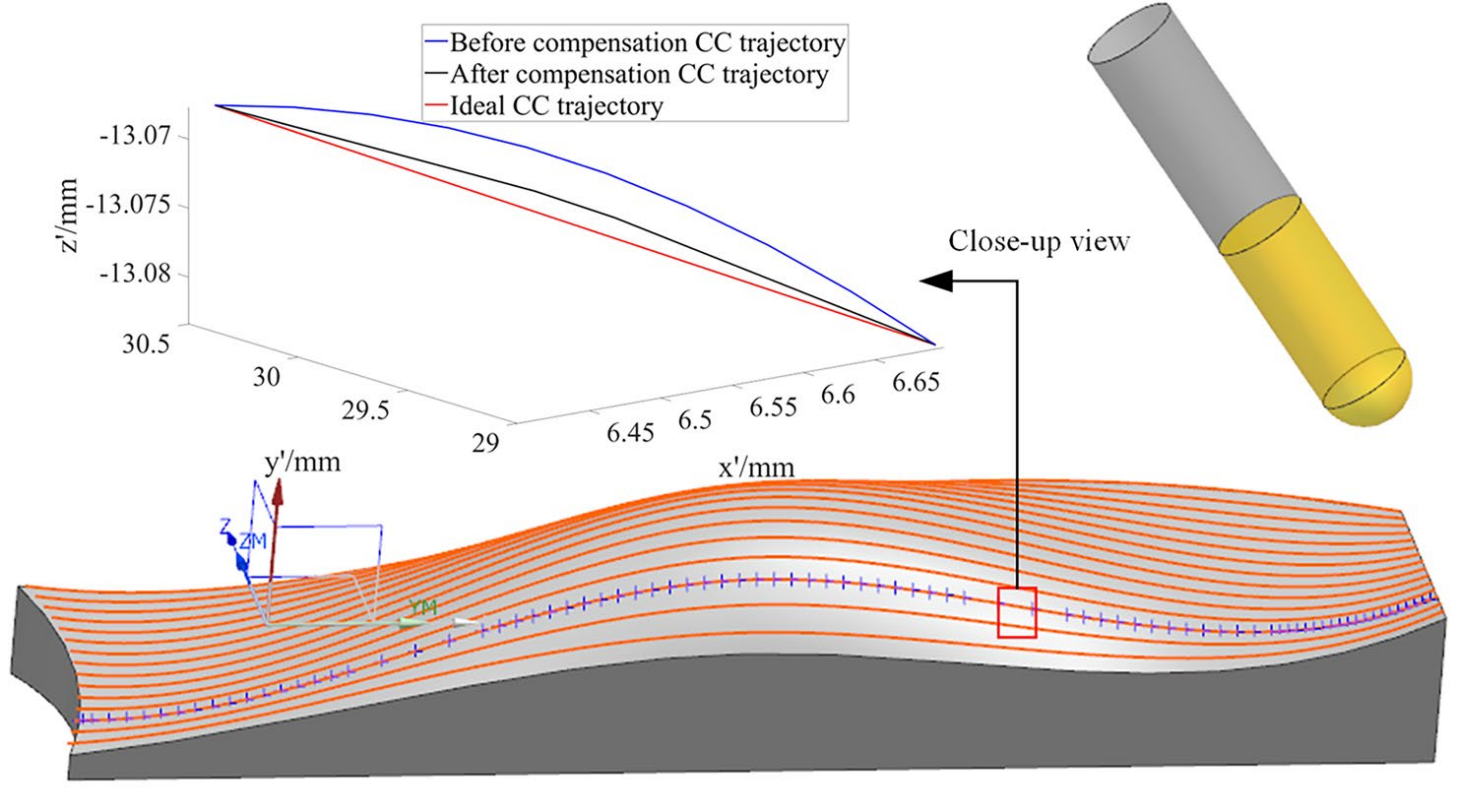
Spatial freeform surface.

Utilizing UG/NX, a spatial freeform surface as shown in Fig. 10 is modeled. In the CAM module, the surface is processed with a discrete tolerance of 3 μm to obtain the required CC point trajectory for machining the surface, as indicated by the red curve in Fig. 10. One of the CC point trajectories (as shown by the blue dot sequence of discrete CC points in Fig. 10) is selected for interpolation calculations and comprehensive nonlinear error compensation and correction. This is done to verify the effectiveness of the methods presented in this paper in reducing contour errors and nonlinear errors in the CC point trajectory. The extracted CC point trajectory consists of 85 points. The corresponding machining cutter location data in the workpiece coordinate system is listed in Table 1. After post-processing calculations, the five-coordinate G01 interpolation instruction data in the machine tool coordinate system can be obtained (Table 2). The complete versions of Tables 1 and 2 are included in the Supplementary material as Supplementary Table S1 and Supplementary Table S2, respectively.

**Table 1:** Machining cutter location data.

No	*x*/mm	*y*/mm	*z*/mm	*I*	*J*	*K*	$x^{\prime}/{\text{mm}}$	$y^{\prime}/{\text{mm}}$	$z^{\prime}/{\text{mm}}$
1	7.0296	45.9771	− 13.3245	0.8183	− 0.5505	0.1651	6.8361	45.6825	− 12.9824
14	5.6728	41.6112	− 13.3236	0.9543	− 0.2989	− 0.0013	5.6279	41.2645	− 12.9786
15	5.6187	41.2620	− 13.3239	0.9596	− 0.2806	− 0.0180	5.5861	40.9136	− 12.9792
84	2.0818	− 10.1172	− 13.3716	0.9790	− 0.1595	− 0.1273	2.1299	− 10.4425	− 13.0319
85	2.0847	− 10.2984	− 13.3722	0.9791	− 0.1592	− 0.1269	2.1299	− 10.4425	− 13.0318

**Table 2:** Five-axis G01 interpolation command data.

No	${\text{X/mm}}$	${\text{Y/mm}}$	${\text{Z/mm}}$	${\uptheta }_{{\text{A}}} {/}^\circ$	${\uptheta }_{{\text{C}}} {/}^\circ$	$X^{\prime}/{\text{mm}}$	$Y^{\prime}/{\text{mm}}$	$Z^{\prime}/{\text{mm}}$
1	42.0720	− 9.8681	− 21.7581	80.4982	56.0704	41.7196	− 9.5313	− 21.6978
14	41.4049	− 13.3328	− 7.0046	90.0753	72.6113	41.0606	− 12.9877	− 6.9443
15	41.1804	− 13.4330	− 5.9477	91.0304	73.6997	40.8369	− 13.0872	− 5.8874
84	− 9.6506	− 12.7941	5.3542	97.3130	80.7437	− 9.9639	− 12.4445	5.4099
85	− 9.8303	− 12.7930	5.3779	97.2930	80.7648	− 9.9653	− 12.4468	5.4019

**Figure 11:**
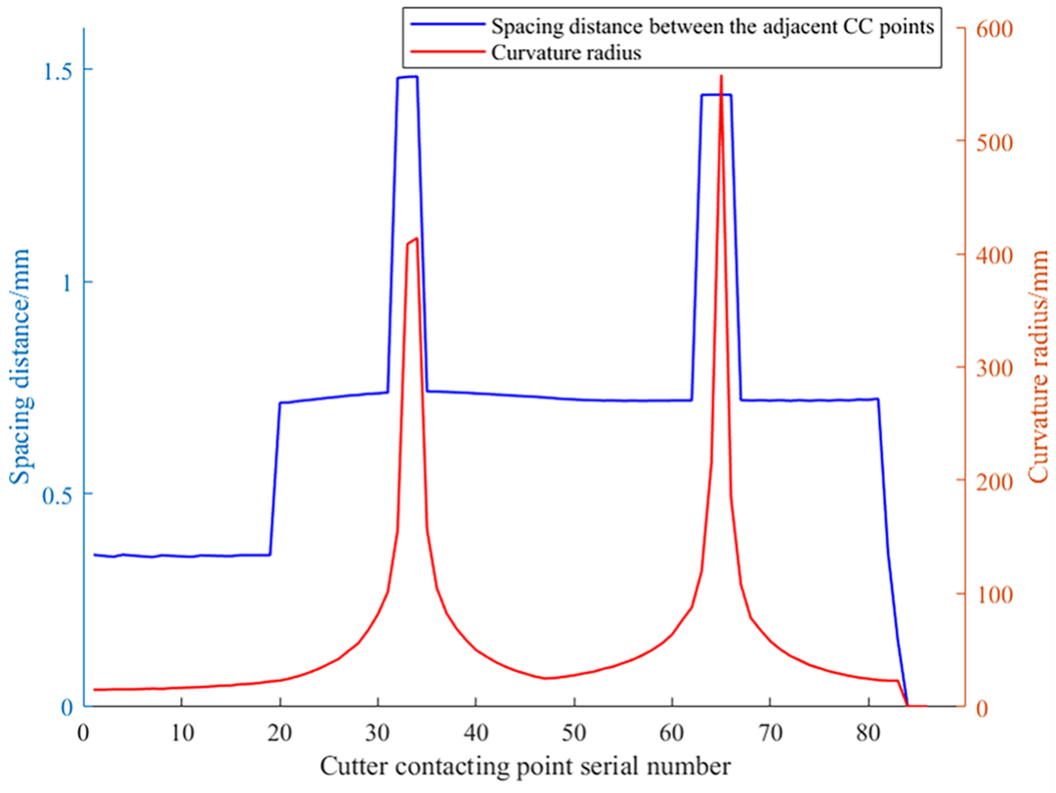
Spacing distance between the adjacent CC points.

**Figure 12:**
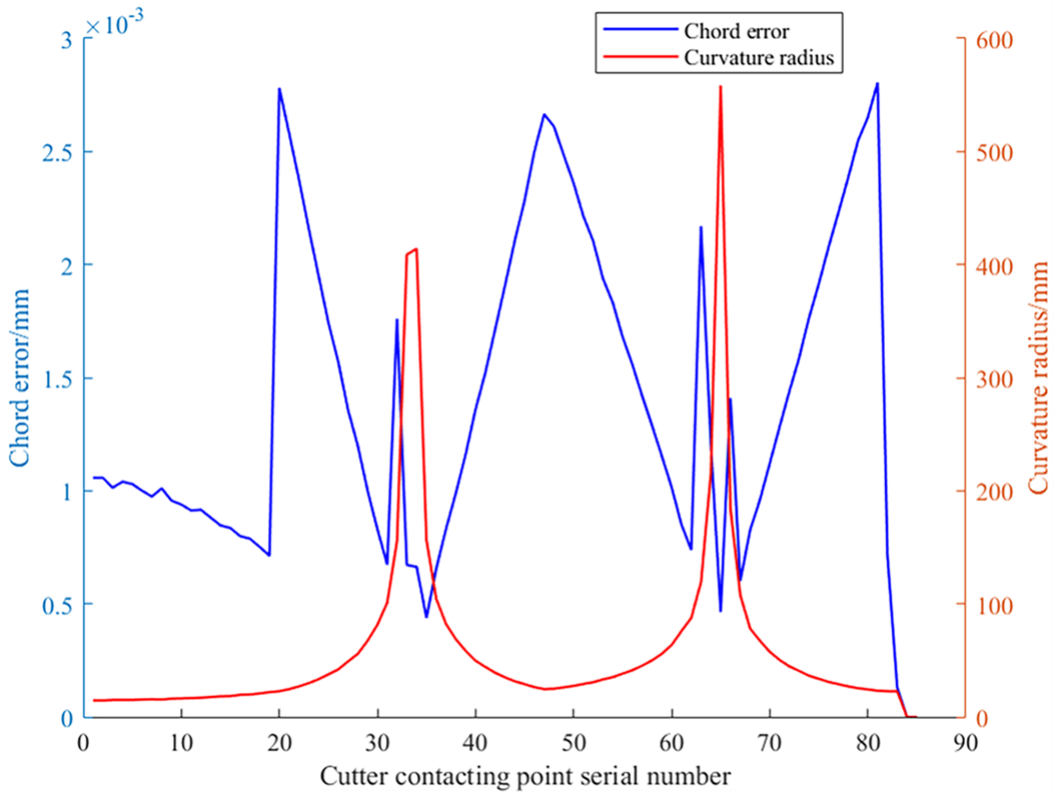
Chord error and curvature radius at the all CC points.

Using Eq. (11), the distance between adjacent CC points on the trajectory shown in Fig. 10 can be calculated. Then, based on Eqs. (9) and (10), the approximate curvature radius and chord error at each CC point on this trajectory can be determined. Based on these calculations, the distance, curvature radius, and chord error data curves at each CC point are plotted respectively as shown in Figs. 11 and 12. It can be observed that to ensure the chord error in approximating the contour of freeform surfaces remains within the tolerance range, at areas with smaller curvature radii, the distance between adjacent CC points is also smaller. A dense CC point approximation approach is adopted to reduce chord error. Conversely, in areas with larger curvature radii, the distance between CC points is increased, utilizing a sparse CC point approximation to improve machining efficiency.

**Figure 13:**
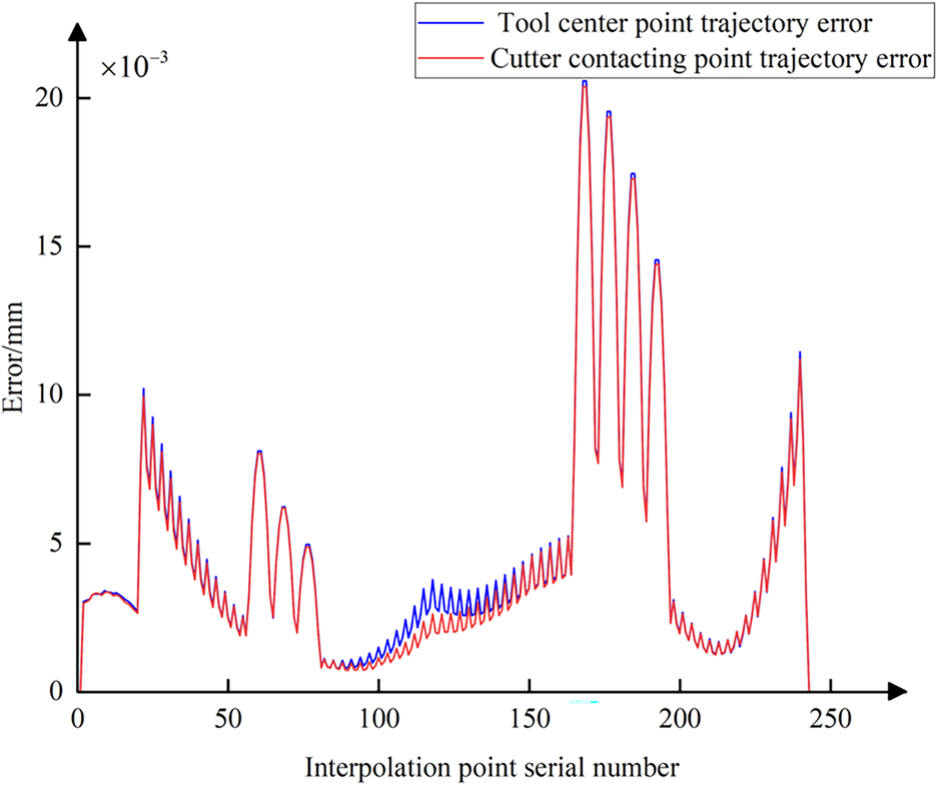
Trajectory error curves of the tool center points and the CC points.

**Figure 14:**
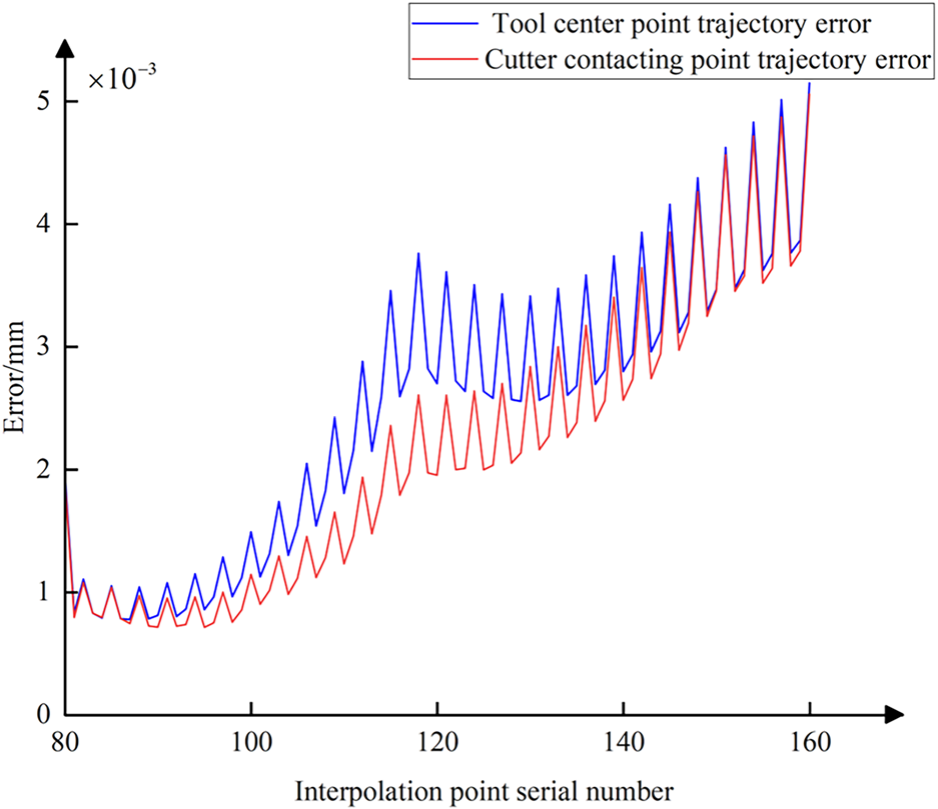
Local amplification of the two trajectory error curves.

Using Eq. (3), the conventional five-axis linear interpolation for the commands in Table 2 is calculated (assuming an interpolation cycle *T* = 2 ms and feed rate *f* = 250 mm/min). Then, based on Eqs. (8) and (19), the nonlinear error in the tool center point position and the positional error at the CC point for each interpolation cycle are calculated. The trajectory error curve is shown in Fig. 13, where the blue curve represents the error of the tool center point and the red curve represents the error of the CC point. It is evident that during the processing of the data in interpolation Table 2, the tool’s swing results in significant positional deviation of the tool center point, with a maximum value reaching 21 μm. With the increase in trajectory error of the tool center point, the positional error of the CC point also grows incrementally. It can be observed that the magnitude of their errors is not significantly different, due to the small distances and angular differences between the start and end points of each interpolation path segment in Table 2. During linear interpolation between these points, the movement between adjacent interpolated CC points can be approximated as micro translational movement, leading to a similarity in the magnitude of errors at the interpolated CC point and the tool center point. However, a detailed observation around interpolated CC point number 120 in Fig. 13 shows that there are still slight differences in their error magnitudes, as depicted in Fig. 14.

**Figure 15:**
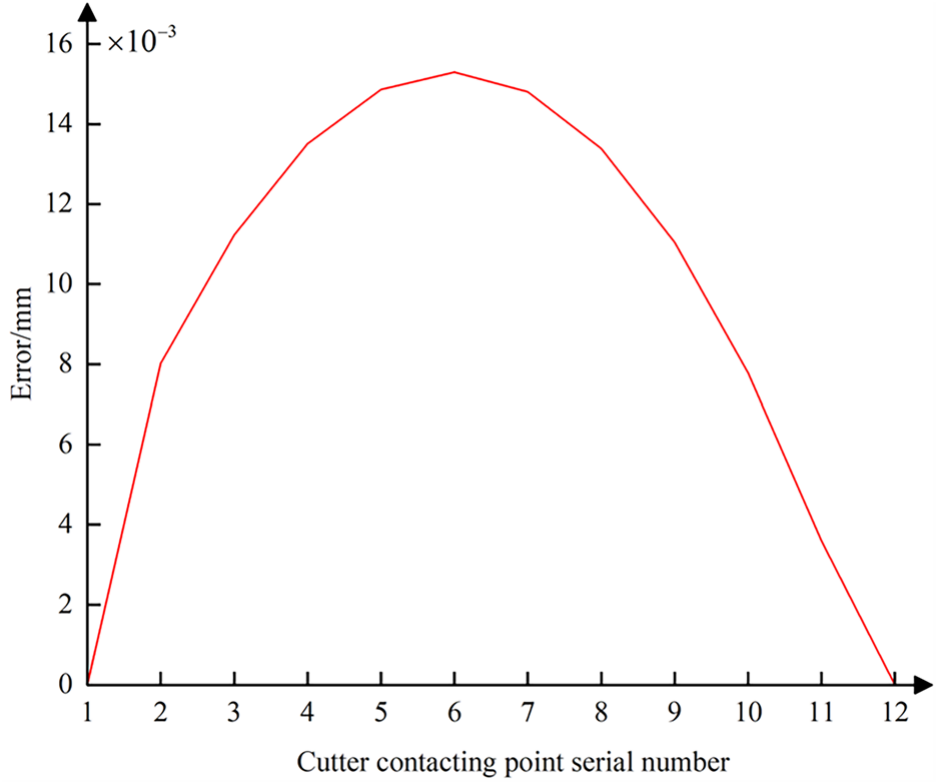
Position error curve of the interpolated CC points between the adjacent CC points with large spacing distance.

To illustrate scenarios where the positional error of the CC point significantly differs from that of the tool center point, data from Table 3 is presented in this paper. The data in Table 3, obtained through post-processing calculations, represents the five-coordinate G01 interpolation instruction data in the machine tool coordinate system. The distance and angular difference between the start and endpoints of the interpolation path segments are 2.162 mm and 5.0008°/8.877°, respectively, which are significantly larger than the data listed in Table 2. Figure 15 shows the positional error data curve of the CC point when the tool center point moves along the ideal tool center point trajectory. Figure 15 indicates that the positional error of the CC point relative to the tool center point can reach a maximum of 15.3 μm. This demonstrates that when the movement between any adjacent interpolated CC points cannot be approximated as microscopic translation, the positional error of the CC point relative to the tool center point is significant and cannot be ignored. Therefore, it is necessary to consider both tool center point trajectory error and CC point trajectory error for compensation and correction when performing nonlinear error compensation.

**Table 3:** Command data for large-spacing path segments.

Parameter	Starting point	Terminal
*X/*mm	35.7172	35.9383
*Y/*mm	1.4329	1.3997
*Z/*mm	25.92	28.0704
*θ*_*A*_*/*(°)	72.5519	67.5511
*θ*_*C*_*/*(°)	88.9829	80.1059

**Figure 16:**
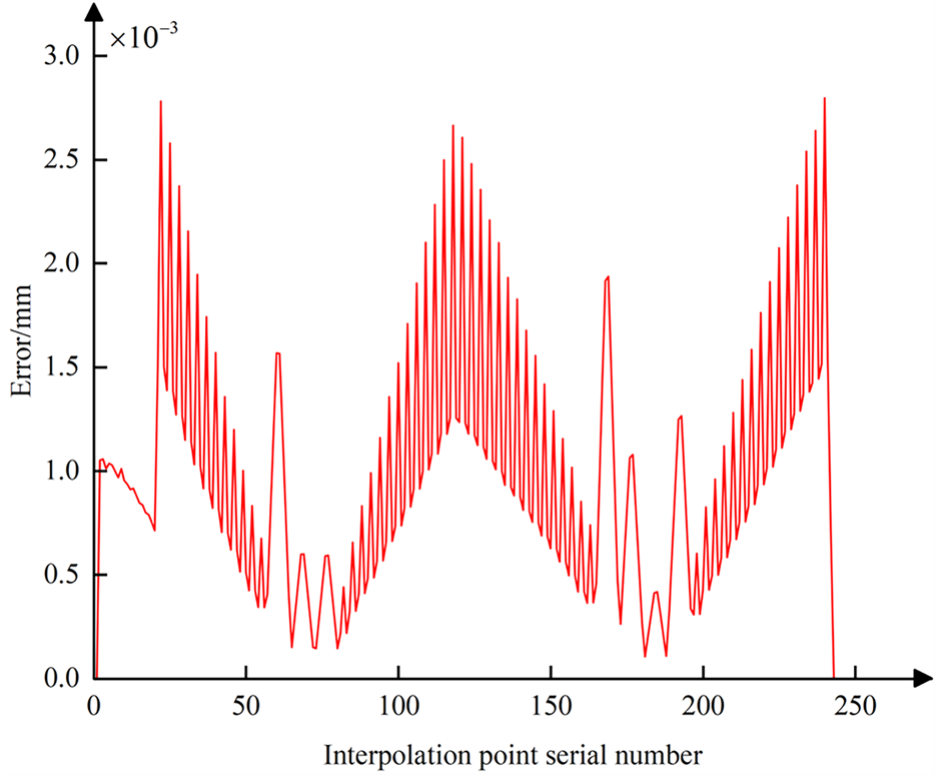
Contour error curve of the interpolated CC points without compensating and correcting.

**Figure 17:**
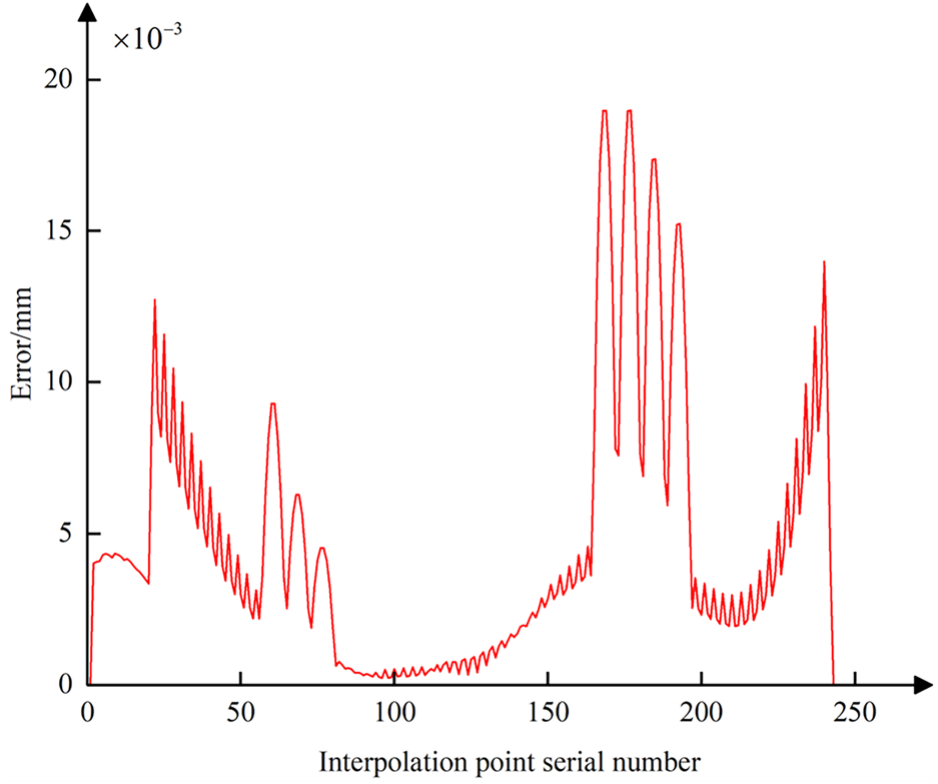
Comprehensive nonlinear error curve of the interpolated CC points without compensating and correcting.

Further, using Eq. (23), the contour error at all interpolated CC points during the conventional five-axis linear interpolation process can be calculated. The data curve of the error is plotted as shown in Fig. 16. It is evident that the contour error at each interpolated CC point fluctuates within the range of the chord error, indicating that the conventional five-axis linear interpolation method cannot adequately address the chord error issue when the CC point trajectory approximates the contour design curve. For subsequent comprehensive compensation and correction of the nonlinear error, Eq. (25) can be used to combine the CC point position error from Fig. 13 and the CC point contour error from Fig. 16. This allows for the calculation of the comprehensive nonlinear error that needs to be compensated and corrected at each interpolated CC point, with its error curve depicted in Fig. 17. The maximum comprehensive nonlinear error of the interpolated CC points can reach 19um. Since the positional error at the interpolated CC points is significantly larger than the contour error, the difference in magnitude between the comprehensive nonlinear error and the positional error is not pronounced after the vector synthesis calculation, with only minor differences.

**Figure 18:**
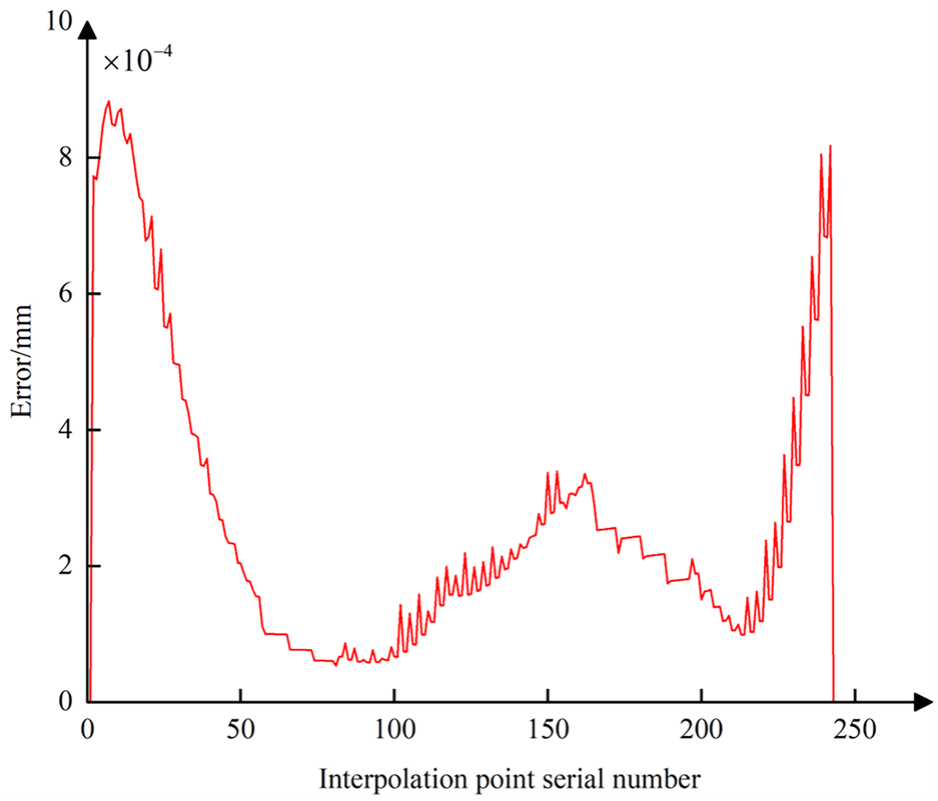
Position error curve of the interpolated CC points using compensation and correction method.

**Figure 19:**
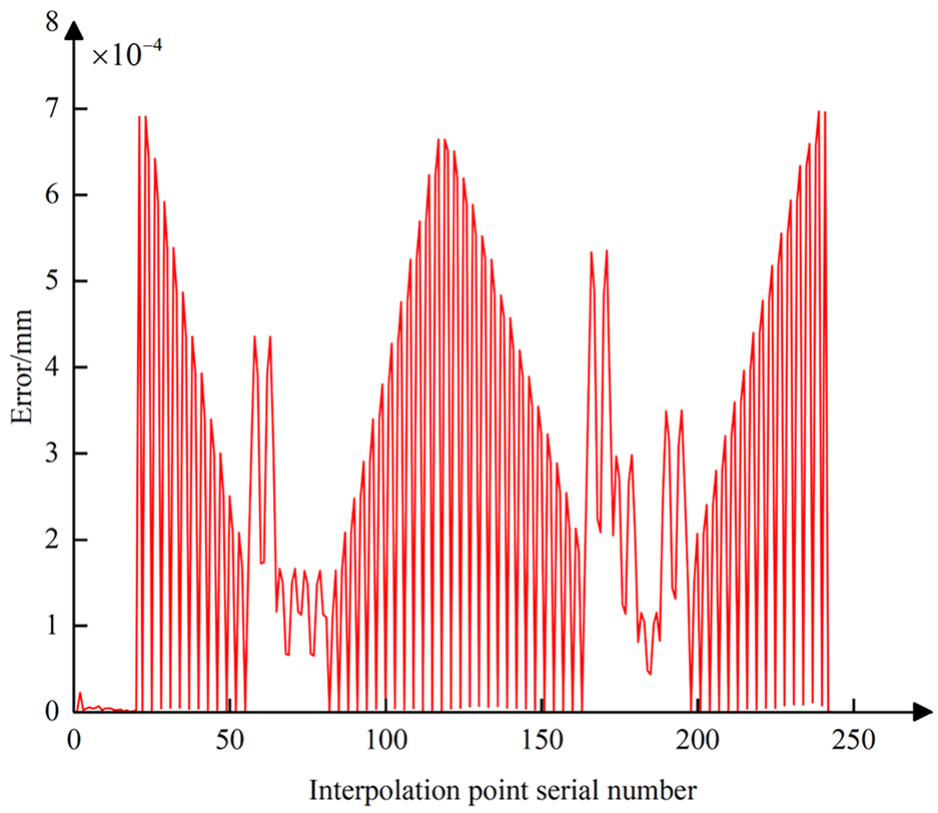
Contour error curve of the interpolated CC points using compensation and correction method.

Following the method of comprehensive compensation and correction of nonlinear errors described in Sect. “Five-axis linear interpolation method incorporating CC points”, Eq. (28) is used to adjust the position of the tool center point while simultaneously readjusting the CC point position. Then, using Eqs. (19) and (23), the curves for the CC point position error and contour error after compensation and correction are plotted (Figs. 18 and 19). A comparison with Figs. 13 and 16 shows that after adjusting the CC point position, the maximum nonlinear error in the CC point trajectory can be reduced from 21 to 0.88 μm, a decrease of 96%; the maximum contour error can also be reduced from 2.8 to 0.69 μm, a reduction of 76%, achieving better machining results than the preset tolerance of 3 μm.

**Figure 20:**
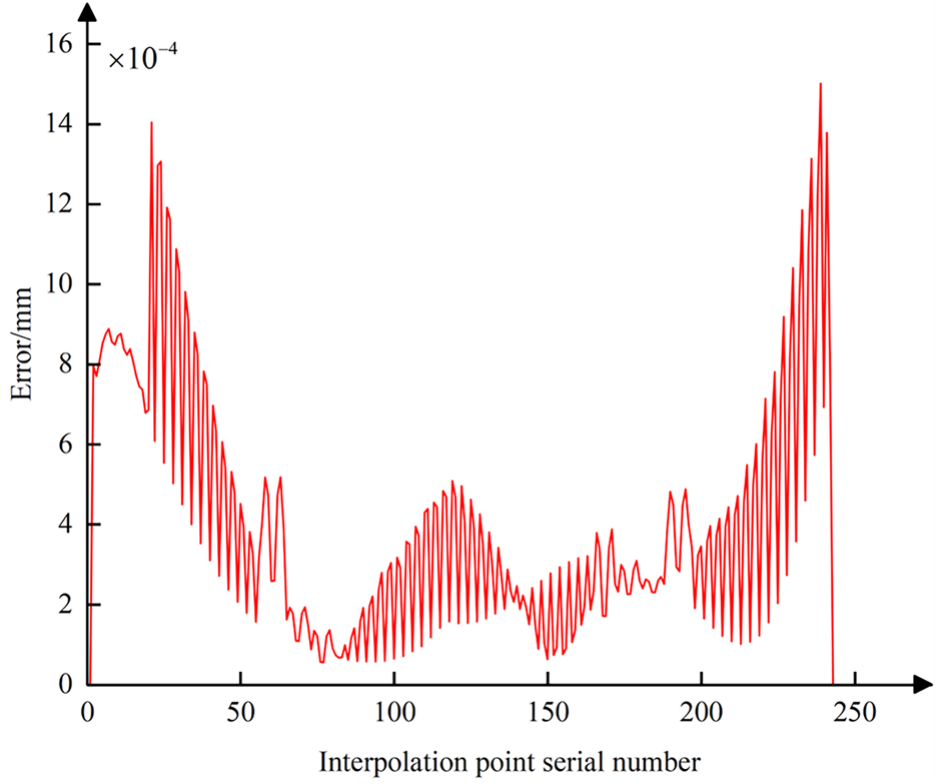
Comprehensive nonlinear error curve after compensation and correction using the method proposed in this paper.

**Figure 21:**
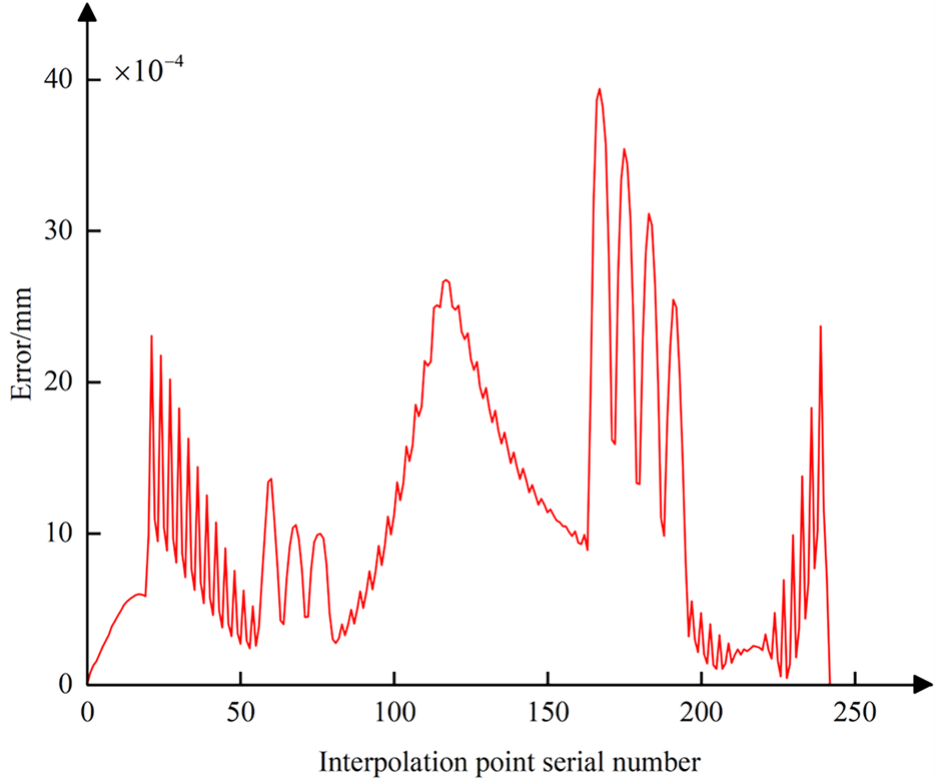
Comprehensive nonlinear error curve after compensation and correction using the method from reference^24^.

Using Eq. (25), the curve for the comprehensive nonlinear error after compensation and correction is plotted (Fig. 20). The maximum comprehensive nonlinear error of the interpolated CC points can reach 1.5 μm. Comparing with Fig. 17, it is evident that after the adjustment of the CC point position, the comprehensive error is reduced from 19 to 1.5 μm, with the reduction also reaching 93%. After zooming in on the trajectory between CC points 32 and 33, the resulting image, shown as Fig. 10, displays the CC point trajectories: the blue line represents the trajectory before compensation, the black line represents the trajectory after compensation, and the red line represents the ideal CC point trajectory. Using the method from reference^24^, we obtained the compensated comprehensive nonlinear error as shown in Fig. 21. This approach only accounted for contour errors, neglecting the positional errors caused by tool deflection. Consequently, the maximum comprehensive nonlinear error after compensation was 3.94 μm, which exceeds the machining tolerance of 3 μm. In contrast, the method proposed in this paper successfully meets the tolerance requirement, with the maximum compensated comprehensive nonlinear error also within limits. A comparison indicates that the method proposed in this paper has good machining accuracy.

**Figure 22:**
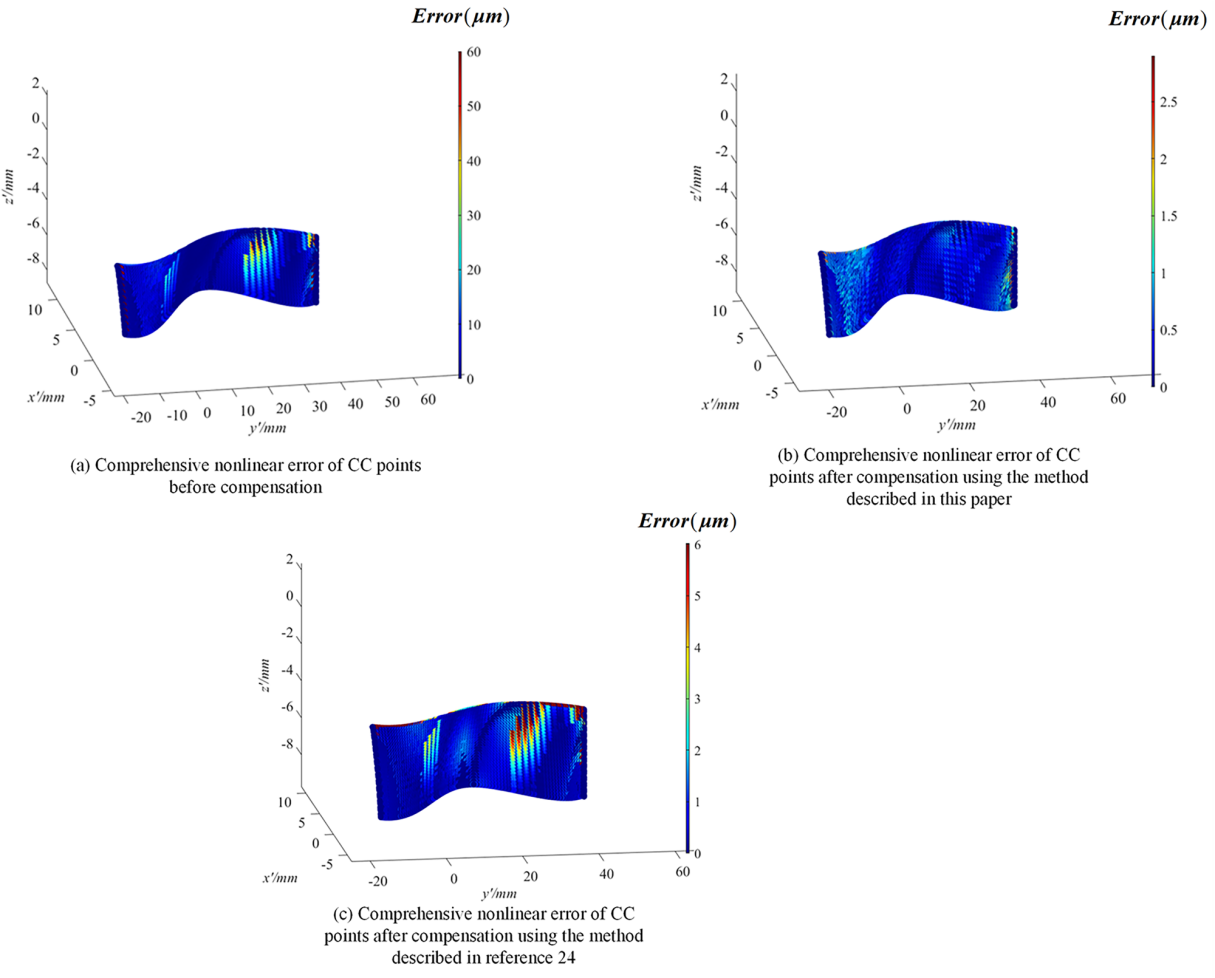
Four-dimensional surface plot before and after compensation.

The above only involves linear interpolation machining for a specific curve. Next, the entire surface shown in Fig. 10 will be processed through linear interpolation. Based on the coordinates of the interpolation points and the error values, a four-dimensional surface will be generated. This surface will be displayed using a mesh grid, and the grid will be colored according to the error values of each point to more intuitively present the effects before and after compensation. Figure 22a shows the comprehensive nonlinear error of CC points before compensation, with a maximum error of 60.5 μm; Fig. 22b shows the comprehensive nonlinear error of the interpolated CC points after compensation and correction using the method described in this paper, with a maximum error of 2.82 μm; Fig. 22c shows the comprehensive nonlinear error of the CC points after correction using the method proposed in reference 24, with a maximum error of 6.02 μm. The experimental results demonstrate that the compensation and correction of CC points proposed in this paper can significantly reduce errors.

## Experiment results

**Figure 23:**
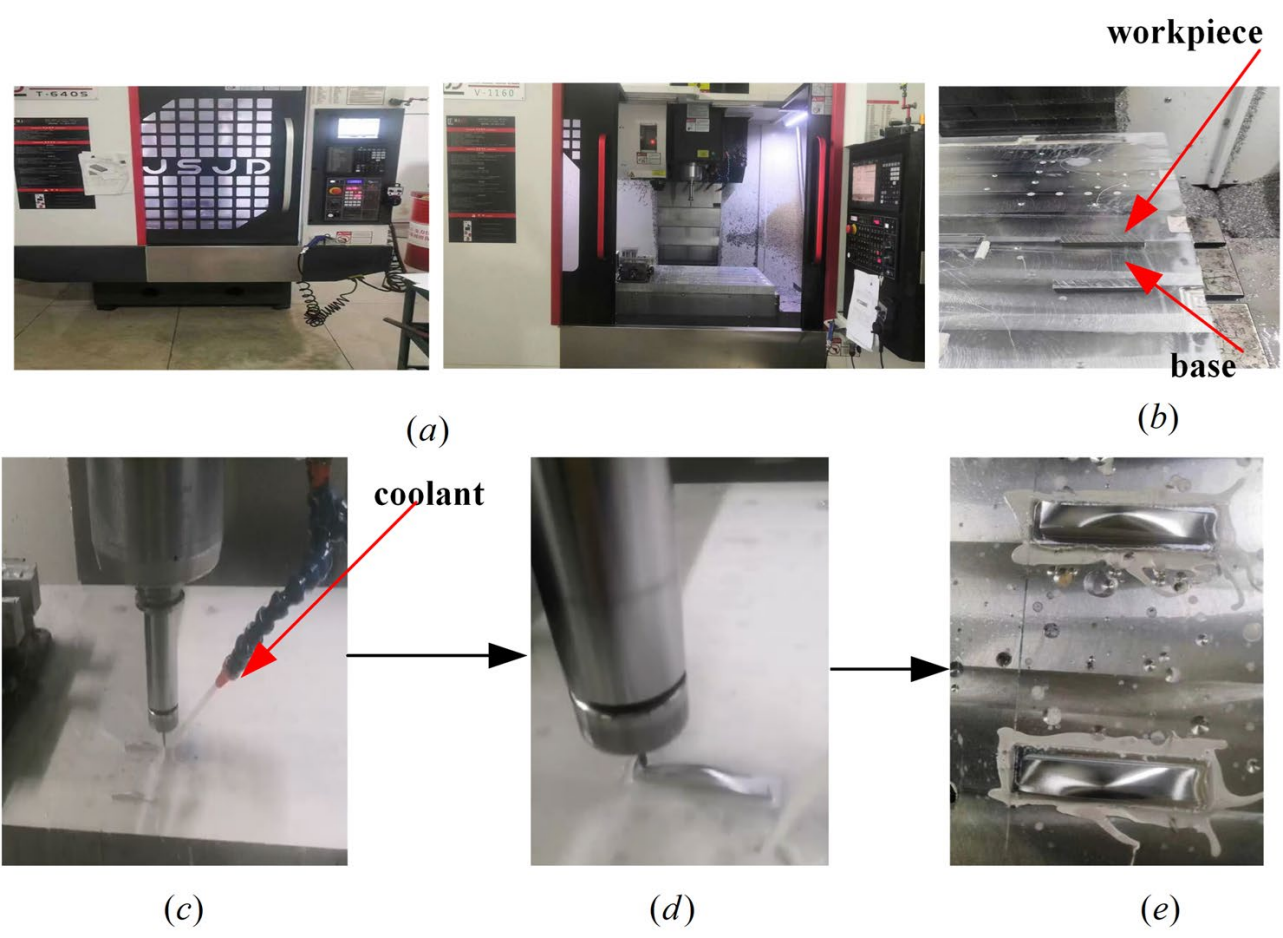
The machining process of spatial freeform surface (**a**) T-6045 and V-1160 machines (**b**) workpiece and base (**c**) rough machining and semi-finishing (**d**) finish machining (**e**) after machining.

To verify the total amount of nonlinear error and compensation methods in this paper, the experiment utilized the spatial freeform surface shown in Fig. 10 for actual machining. The processing was conducted on T-6045 and V-1160 machines using the FANUC-0i MD system, comprising three stages: rough machining, semi-finishing, and finishing. A 65 mm*15 mm*15 mm aluminum 6061 workpiece was used. Initial rough machining and semi-finishing were performed on the T-6045 machine using a 10 mm diameter flat end mill for roughing, followed by a 4 mm diameter flat end mill and ball end mill for semi-finishing to achieve the basic shape. Finally, finishing was carried out on the V-1160 machine, using the method proposed in this paper as well as the traditional method without compensation for comparison. A 4 mm diameter ball end mill was used with an interpolation cycle *T* = 2 ms and a feed rate *f* = 250 mm/min. The machining process are shown in Fig. 23.

**Figure 24:**
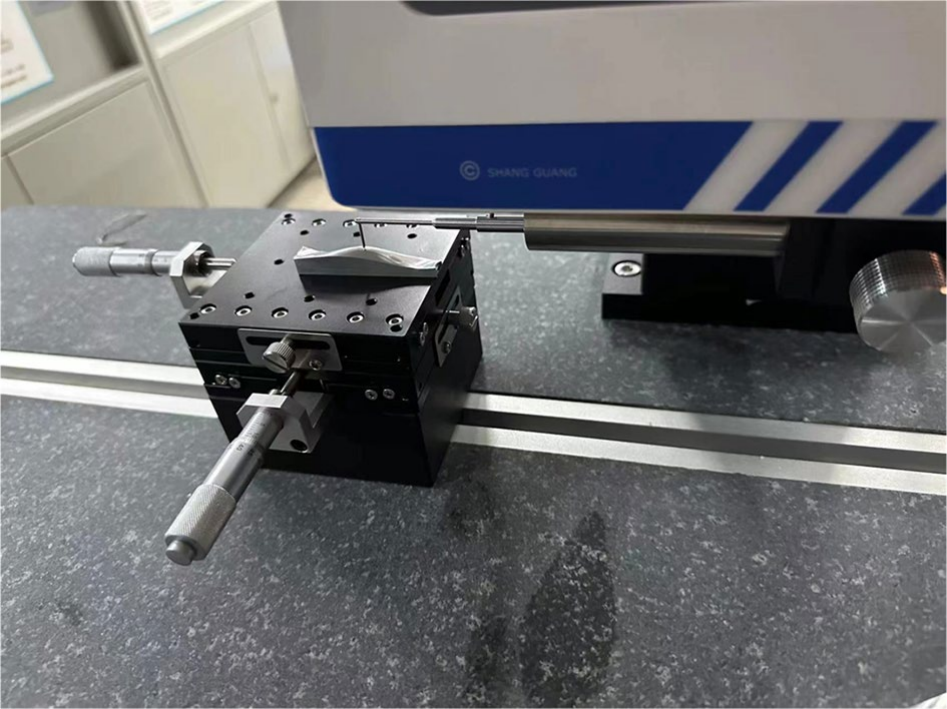
Measuring the roughness of machined parts with the SRA series surface roughness tester.

**Figure 25:**
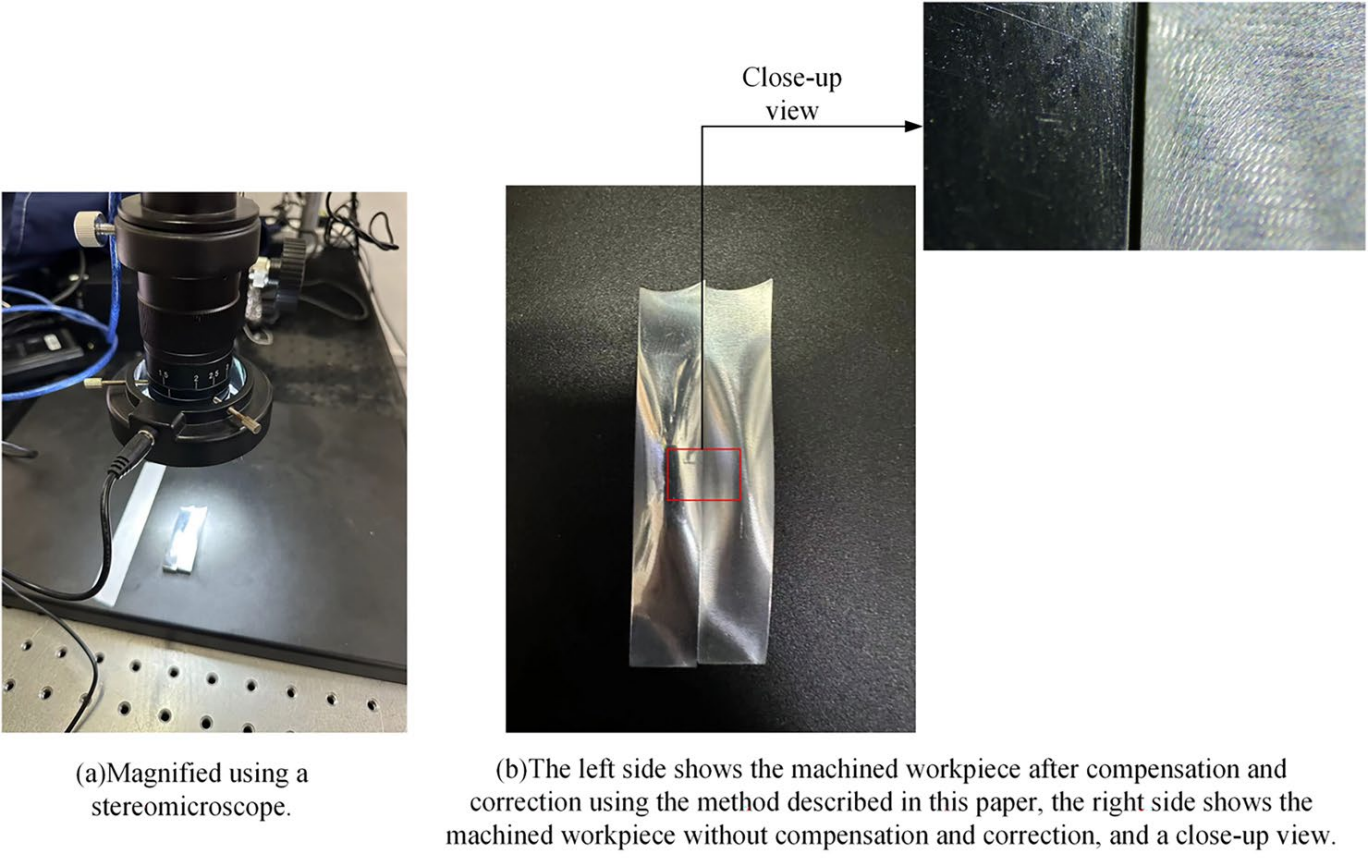
Comparison of machined workpieces before and after compensation and correction.

As shown in Fig. 24, the surface roughness of the machined surface was measured using the SRA series surface roughness tester. The machining result without compensation, as shown in Table 4, had an average surface roughness of 1.133 μm and a maximum surface roughness of 6.667 μm. After machining with the nonlinear error comprehensive compensation method proposed in this paper, the surface roughness obtained is shown in Table 4. The average surface roughness was reduced to 0.220 μm, and the maximum surface roughness was decreased to 1.240 μm. In comparison, the compensation and correction methods adopted in this paper significantly improved the machining effect and quality of the parts. Figure 25 shows a comparative diagram of the machined workpieces before and after compensation and correction. The close-up view is a magnified image using a stereomicroscope, which also demonstrates that the method proposed in this paper significantly improved the machining precision of the parts.

**Table 4:** Comparison of the roughness of machined parts before and after compensation and correction.

		Before compensation and correction	After compensation and correction
Actual machining of spatial freeform surfaces	Average surface roughness	1.133 μm	0.220 μm
	Maximum surface roughness	6.667 μm	1.240 μm

## Conclusions

1. The mechanism of chord error generation at interpolated CC points is analyzed in this paper, and a method for calculating the chord error of interpolation points by using an approximate circular arc approach is proposed; It also analyzes the mechanisms of trajectory errors at interpolated tool center points and positional errors at CC points, proposing a method to determine the actual CC points based on the current actual interpolated tool center points.
2. The introduction of contour error combines the CC point contour error with the positional error of the CC point during interpolation into a comprehensive nonlinear error. A method for calculating the magnitude and compensation direction of this error has been proposed and implemented, resulting in new coordinates for the interpolated tool center point.
3. Using Matlab, the curves of comprehensive nonlinear error data before and after correction were plotted. After compensation and correction, the contour error was reduced by 76%, and the nonlinear error of the CC point trajectory was reduced to below 0.88 μm. The comprehensive nonlinear error of the CC point trajectory decreased from 19 to 1.5 μm, a reduction of 93%. And the entire surface underwent simulation calculations, with the nonlinear error after compensation also meeting the machining tolerance of 3 μm. Finally, using this comprehensive compensation method for nonlinear errors in actual machining, the average surface roughness was reduced from 1.133 to 0.220 μm, and the maximum surface roughness was reduced from 6.667 to 1.240 μm. This method significantly reduces the interpolation error of the CC point position and the comprehensive nonlinear error, and it has important practical value in improving the accuracy of CC point trajectory control in five-axis CNC machining.
4. However, the main focus of this study is on the impact of CC point trajectory error on the precision of five-axis CNC machining. Additionally, the influence of other error sources on the precision of five-axis CNC machining will be analyzed and discussed in future research.

### Supplementary Information

Supplementary Information.

## Data Availability

Main data generated or analyzed during this study are included in this article. All data in this article are available from the corresponding author on reasonable request.

## List of symbols

***a***: The vector pointing from the initial tool center point position vector to the terminal tool center point position vector. *a* = *O*_*e*_ − *O*_*s*_
***b***: The vector pointing from the initial tool center point position vector to the *i*-th interpolated tool center point position vector. *b* = *O*_*i*_ − *O*_*s*_
*D*: The total length of the path segment to be interpolated.
***D***_*s*_: The unit vector in the direction of the tool axis at the start point.
***D***_*e*_: The unit vector in the direction of the tool axis at the end point.
***D***_*i*_: The unit vector in the direction of the tool axis at the *i-*th interpolation point under ideal conditions.
$\user2{D^{\prime}}_{i}$: The unit vector in the direction of the tool axis at the *i*-th interpolation point under actual conditions.
*f*: Feed rate
*L*: Swing tool length
*n*: The total number of interpolation cycles required between the start and endpoint
***O***_*s*_: The position vector of the initial tool center point in the workpiece coordinate system.
***O***_*e*_: The position vector of the terminal tool center point in the workpiece coordinate system.
***O***_*i*_: The ideal position vector of the *i-*th interpolated tool center point in the workpiece coordinate system.
$\user2{O^{\prime}}_{i}$: The actual position vector of the *i-*th interpolated tool center point in the workpiece coordinate system.
*T*: The CNC system interpolation cycle
***T***_*s*_: The position vector of the initial tool axis point.
***T***_*e*_: The position vector of the terminal tool axis point.
***T***_*i*_: The ideal position vector of the *i*-th interpolated tool axis point
$\user2{T^{\prime}}_{i}$: The actual position vector of the *i*-th interpolated tool axis point
$(X_{s} ,Y_{s} ,Z_{s} ,\theta_{As} ,\theta_{Cs} )$: The start point of the path segment in the machine tool coordinate system
$(X_{e} ,Y_{e} ,Z_{e} ,\theta_{Ae} ,\theta_{Ce} )$: The end point of the path segment in the machine tool coordinate system
$(x_{i} {\text{,y}}_{i} {\text{,z}}_{i} )$: The target motion position coordinates of each translational axis of the machine tool for the *i*-th interpolation cycle
$(X_{i} {\text{,Y}}_{i} {\text{,Z}}_{i} )$: The *i*-th interpolated tool center point
$\left( {\theta_{Ai} ,\theta_{Ci} } \right)$: The target motion position coordinates for the A and C rotation axes
$\sigma$: The nonlinear error in the tool center point trajectory during five-axis linear interpolation.
$\sigma_{i}$: The nonlinear error at the *i*-th interpolated tool center point
