# Supplementary material for: An integrated method for compensating and correcting nonlinear error in five-axis machining utilizing cutter contacting point data
Source: Sci Rep. 2024 Apr 16;14:8763. doi: 10.1038/s41598-024-59458-w (PMC11021413; doi:10.1038/s41598-024-59458-w)
Supplement: Supplementary file 1 — Supplementary Information. [file 41598_2024_59458_MOESM1_ESM.docx]

Supplementary Table S1 Machining cutter location data

| No. | *x/*mm | *y/*mm | *z/*mm | *I* | *J* | *K* | *x'/*mm | *y'/*mm | *z'/*mm |
| --- | --- | --- | --- | --- | --- | --- | --- | --- | --- |
| 1 | 7.0296 | 45.9771 | -13.3245 | 0.8183 | -0.5505 | 0.1651 | 6.8361 | 45.6825 | -12.9824 |
| 2 | 6.8803 | 45.6551 | -13.3245 | 0.8323 | -0.5315 | 0.1573 | 6.6963 | 45.3550 | -12.9821 |
| 3 | 6.7393 | 45.3320 | -13.3245 | 0.8458 | -0.5124 | 0.1485 | 6.5653 | 45.0265 | -12.9816 |
| 4 | 6.6064 | 45.0078 | -13.3244 | 0.8588 | -0.4932 | 0.1388 | 6.4427 | 44.6971 | -12.9811 |
| 5 | 6.4790 | 44.6761 | -13.3243 | 0.8714 | -0.4735 | 0.1280 | 6.3262 | 44.3604 | -12.9805 |
| 6 | 6.3598 | 44.3435 | -13.3242 | 0.8835 | -0.4538 | 0.1163 | 6.2182 | 44.0230 | -12.9800 |
| 7 | 6.2486 | 44.0098 | -13.3240 | 0.8948 | -0.4341 | 0.1039 | 6.1184 | 43.6849 | -12.9795 |
| 8 | 6.1452 | 43.6753 | -13.3238 | 0.9055 | -0.4145 | 0.0907 | 6.0266 | 43.3463 | -12.9790 |
| 9 | 6.0480 | 43.3346 | -13.3237 | 0.9156 | -0.3947 | 0.0767 | 5.9415 | 43.0017 | -12.9786 |
| 10 | 5.9585 | 42.9931 | -13.3235 | 0.9249 | -0.3751 | 0.0620 | 5.8642 | 42.6567 | -12.9783 |
| 11 | 5.8766 | 42.6507 | -13.3234 | 0.9334 | -0.3557 | 0.0469 | 5.7945 | 42.3112 | -12.9781 |
| 12 | 5.8020 | 42.3076 | -13.3234 | 0.9412 | -0.3365 | 0.0313 | 5.7322 | 41.9654 | -12.9781 |
| 13 | 5.7338 | 41.9598 | -13.3235 | 0.9481 | -0.3175 | 0.0151 | 5.6765 | 41.6151 | -12.9783 |
| 14 | 5.6728 | 41.6112 | -13.3236 | 0.9543 | -0.2989 | -0.0013 | 5.6279 | 41.2645 | -12.9786 |
| 15 | 5.6187 | 41.2620 | -13.3239 | 0.9596 | -0.2806 | -0.0180 | 5.5861 | 40.9136 | -12.9792 |
| 16 | 5.5714 | 40.9121 | -13.3242 | 0.9642 | -0.2629 | -0.0348 | 5.5510 | 40.5624 | -12.9799 |
| 17 | 5.5302 | 40.5589 | -13.3247 | 0.9680 | -0.2455 | -0.0519 | 5.5220 | 40.2082 | -12.9809 |
| 18 | 5.4956 | 40.2050 | -13.3253 | 0.9711 | -0.2286 | -0.0689 | 5.4993 | 39.8537 | -12.9821 |
| 19 | 5.4671 | 39.8506 | -13.3260 | 0.9734 | -0.2123 | -0.0860 | 5.4826 | 39.4989 | -12.9836 |
| 20 | 5.4446 | 39.4957 | -13.3269 | 0.9751 | -0.1966 | -0.1030 | 5.4716 | 39.1439 | -12.9852 |
| 21 | 5.4168 | 38.7810 | -13.3290 | 0.9765 | -0.1669 | -0.1367 | 5.4661 | 38.4297 | -12.9893 |
| 22 | 5.4108 | 38.0643 | -13.3317 | 0.9755 | -0.1398 | -0.1696 | 5.4812 | 37.7146 | -12.9941 |
| 23 | 5.4250 | 37.3442 | -13.3349 | 0.9727 | -0.1152 | -0.2016 | 5.5153 | 36.9967 | -12.9997 |
| 24 | 5.4578 | 36.6224 | -13.3385 | 0.9681 | -0.0932 | -0.2324 | 5.5667 | 36.2777 | -13.0060 |
| 25 | 5.5076 | 35.8988 | -13.3425 | 0.9623 | -0.0738 | -0.2619 | 5.6338 | 35.5573 | -13.0129 |
| 26 | 5.5728 | 35.1737 | -13.3468 | 0.9553 | -0.0568 | -0.2900 | 5.7150 | 34.8356 | -13.0202 |
| 27 | 5.6520 | 34.4479 | -13.3514 | 0.9476 | -0.0423 | -0.3168 | 5.8089 | 34.1133 | -13.0278 |
| 28 | 5.7436 | 33.7207 | -13.3561 | 0.9392 | -0.0301 | -0.3421 | 5.9139 | 33.3897 | -13.0357 |
| 29 | 5.8460 | 32.9936 | -13.3609 | 0.9304 | -0.0200 | -0.3660 | 6.0286 | 32.6660 | -13.0437 |
| 30 | 5.9580 | 32.2653 | -13.3657 | 0.9213 | -0.0120 | -0.3886 | 6.1516 | 31.9410 | -13.0518 |
| 31 | 6.0777 | 31.5374 | -13.3706 | 0.9122 | -0.0060 | -0.4098 | 6.2813 | 31.2162 | -13.0598 |
| 32 | 6.2042 | 30.8087 | -13.3754 | 0.9030 | -0.0019 | -0.4297 | 6.4167 | 30.4901 | -13.0678 |
| 33 | 6.4710 | 29.3517 | -13.3848 | 0.8849 | 0.0012 | -0.4658 | 6.6983 | 29.0375 | -13.0834 |
| 34 | 6.7474 | 27.8949 | -13.3936 | 0.8676 | -0.0022 | -0.4973 | 6.9856 | 27.5832 | -13.0980 |
| 35 | 7.0225 | 26.4385 | -13.4018 | 0.8514 | -0.0114 | -0.5243 | 7.2679 | 26.1274 | -13.1115 |
| 36 | 7.1562 | 25.7106 | -13.4055 | 0.8439 | -0.0182 | -0.5363 | 7.4038 | 25.3990 | -13.1178 |
| 37 | 7.2855 | 24.9827 | -13.4091 | 0.8366 | -0.0264 | -0.5471 | 7.5345 | 24.6700 | -13.1236 |
| 38 | 7.4092 | 24.2550 | -13.4124 | 0.8298 | -0.0359 | -0.5570 | 7.6585 | 23.9407 | -13.1290 |
| 39 | 7.5258 | 23.5275 | -13.4155 | 0.8233 | -0.0468 | -0.5657 | 7.7745 | 23.2112 | -13.1340 |
| 40 | 7.6340 | 22.8002 | -13.4184 | 0.8172 | -0.0591 | -0.5734 | 7.8812 | 22.4811 | -13.1384 |
| 41 | 7.7324 | 22.0733 | -13.4209 | 0.8114 | -0.0728 | -0.5799 | 7.9769 | 21.7511 | -13.1423 |
| 42 | 7.8196 | 21.3465 | -13.4231 | 0.8061 | -0.0879 | -0.5852 | 8.0604 | 21.0206 | -13.1456 |
| 43 | 7.8942 | 20.6204 | -13.4250 | 0.8012 | -0.1045 | -0.5892 | 8.1301 | 20.2903 | -13.1481 |
| 44 | 7.9548 | 19.8945 | -13.4266 | 0.7966 | -0.1227 | -0.5919 | 8.1845 | 19.5599 | -13.1500 |
| 45 | 7.9999 | 19.1695 | -13.4277 | 0.7925 | -0.1425 | -0.5930 | 8.2220 | 18.8300 | -13.1509 |
| 46 | 8.0281 | 18.4453 | -13.4284 | 0.7888 | -0.1640 | -0.5924 | 8.2412 | 18.1006 | -13.1510 |
| 47 | 8.0378 | 17.7222 | -13.4287 | 0.7855 | -0.1872 | -0.5899 | 8.2404 | 17.3722 | -13.1500 |
| 48 | 8.0274 | 17.0008 | -13.4284 | 0.7829 | -0.2112 | -0.5853 | 8.2183 | 16.6457 | -13.1479 |
| 49 | 7.9965 | 16.2815 | -13.4276 | 0.7813 | -0.2341 | -0.5786 | 8.1754 | 15.9220 | -13.1448 |
| 50 | 7.9463 | 15.5649 | -13.4263 | 0.7808 | -0.2561 | -0.5699 | 8.1128 | 15.2016 | -13.1407 |
| 51 | 7.8777 | 14.8504 | -13.4245 | 0.7814 | -0.2772 | -0.5591 | 8.0315 | 14.4840 | -13.1358 |
| 52 | 7.7919 | 14.1389 | -13.4223 | 0.7829 | -0.2974 | -0.5464 | 7.9328 | 13.7701 | -13.1301 |
| 53 | 7.6898 | 13.4294 | -13.4197 | 0.7854 | -0.3168 | -0.5318 | 7.8175 | 13.0589 | -13.1237 |
| 54 | 7.5727 | 12.7230 | -13.4168 | 0.7888 | -0.3354 | -0.5151 | 7.6871 | 12.3514 | -13.1167 |
| 55 | 7.4413 | 12.0185 | -13.4135 | 0.7929 | -0.3532 | -0.4966 | 7.5423 | 11.6465 | -13.1091 |
| 56 | 7.2971 | 11.3169 | -13.4099 | 0.7977 | -0.3700 | -0.4761 | 7.3845 | 10.9451 | -13.1011 |
| 57 | 7.1407 | 10.6172 | -13.4060 | 0.8032 | -0.3859 | -0.4539 | 7.2145 | 10.2462 | -13.0928 |
| 58 | 6.9734 | 9.9202 | -13.4019 | 0.8091 | -0.4009 | -0.4298 | 7.0336 | 9.5505 | -13.0843 |
| 59 | 6.7961 | 9.2248 | -13.3977 | 0.8153 | -0.4147 | -0.4041 | 6.8426 | 8.8569 | -13.0757 |
| 60 | 6.6100 | 8.5318 | -13.3935 | 0.8217 | -0.4274 | -0.3769 | 6.6428 | 8.1661 | -13.0672 |
| 61 | 6.4158 | 7.8402 | -13.3892 | 0.8283 | -0.4389 | -0.3484 | 6.4351 | 7.4772 | -13.0590 |
| 62 | 6.2149 | 7.1505 | -13.3849 | 0.8348 | -0.4490 | -0.3188 | 6.2209 | 6.7903 | -13.0511 |
| 63 | 6.0080 | 6.4620 | -13.3808 | 0.8412 | -0.4576 | -0.2883 | 6.0010 | 6.1049 | -13.0437 |
| 64 | 5.5807 | 5.0884 | -13.3734 | 0.8533 | -0.4701 | -0.2258 | 5.5489 | 4.7379 | -13.0308 |
| 65 | 5.1423 | 3.7175 | -13.3674 | 0.8642 | -0.4758 | -0.1635 | 5.0881 | 3.3734 | -13.0212 |
| 66 | 4.7014 | 2.3470 | -13.3634 | 0.8741 | -0.4745 | -0.1042 | 4.6281 | 2.0084 | -13.0152 |
| 67 | 4.2664 | 0.9746 | -13.3614 | 0.8833 | -0.4660 | -0.0508 | 4.1784 | 0.6402 | -13.0125 |
| 68 | 4.0538 | 0.2865 | -13.3610 | 0.8880 | -0.4591 | -0.0272 | 3.9602 | -0.0465 | -13.0123 |
| 69 | 3.8462 | -0.4021 | -13.3611 | 0.8928 | -0.4505 | -0.0061 | 3.7486 | -0.7342 | -13.0126 |
| 70 | 3.6442 | -1.0933 | -13.3615 | 0.8978 | -0.4401 | 0.0122 | 3.5441 | -1.4249 | -13.0133 |
| 71 | 3.4495 | -1.7853 | -13.3621 | 0.9033 | -0.4282 | 0.0272 | 3.3485 | -2.1171 | -13.0144 |
| 72 | 3.2626 | -2.4805 | -13.3629 | 0.9092 | -0.4145 | 0.0388 | 3.1625 | -2.8130 | -13.0155 |
| 73 | 3.0852 | -3.1768 | -13.3637 | 0.9157 | -0.3992 | 0.0465 | 2.9880 | -3.5105 | -13.0167 |
| 74 | 2.9178 | -3.8769 | -13.3645 | 0.9228 | -0.3821 | 0.0500 | 2.8255 | -4.2123 | -13.0177 |
| 75 | 2.7623 | -4.5783 | -13.3652 | 0.9303 | -0.3634 | 0.0490 | 2.6771 | -4.9158 | -13.0187 |
| 76 | 2.6193 | -5.2839 | -13.3658 | 0.9384 | -0.3428 | 0.0433 | 2.5434 | -5.6237 | -13.0194 |
| 77 | 2.4904 | -5.9910 | -13.3663 | 0.9467 | -0.3205 | 0.0326 | 2.4263 | -6.3333 | -13.0201 |
| 78 | 2.3764 | -6.7024 | -13.3668 | 0.9550 | -0.2963 | 0.0167 | 2.3265 | -7.0474 | -13.0207 |
| 79 | 2.2791 | -7.4153 | -13.3672 | 0.9628 | -0.2703 | -0.0044 | 2.2458 | -7.7627 | -13.0216 |
| 80 | 2.1993 | -8.1326 | -13.3678 | 0.9697 | -0.2424 | -0.0309 | 2.1851 | -8.4821 | -13.0230 |
| 81 | 2.1384 | -8.8513 | -13.3687 | 0.9750 | -0.2131 | -0.0625 | 2.1456 | -9.2023 | -13.0251 |
| 82 | 2.0976 | -9.5742 | -13.3701 | 0.9783 | -0.1822 | -0.0988 | 2.1283 | -9.9258 | -13.0285 |
| 83 | 2.0851 | -9.9362 | -13.3710 | 0.9789 | -0.1664 | -0.1186 | 2.1282 | -10.2877 | -13.0308 |
| 84 | 2.0818 | -10.1172 | -13.3716 | 0.9790 | -0.1595 | -0.1273 | 2.1299 | -10.4425 | -13.0319 |
| 85 | 2.0847 | -10.2984 | -13.3722 | 0.9791 | -0.1592 | -0.1269 | 2.1299 | -10.4425 | -13.0318 |

Supplementary Table S2 Five-axis G01 interpolation command data

| No. | *X/*mm | *Y/*mm | *Z/*mm |    \|  \| \| --- \| |    \|  \| \| --- \| | *X'/*mm | *Y'/*mm | *Z'/*mm |
| --- | --- | --- | --- | --- | --- | --- | --- | --- | --- | --- |
| 1 | 42.0720 | -9.8681 | -21.7581 | 80.4982 | 56.0704 | 41.7196 | -9.5313 | -21.6978 |
| 2 | 42.1809 | -10.2055 | -20.6370 | 80.9503 | 57.4359 | 41.8290 | -9.8684 | -20.5768 |
| 3 | 42.2635 | -10.5441 | -19.5081 | 81.4589 | 58.7904 | 41.9121 | -10.2064 | -19.4478 |
| 4 | 42.3194 | -10.8792 | -18.3739 | 82.0208 | 60.1310 | 41.9684 | -10.5410 | -18.3136 |
| 5 | 42.3485 | -11.2130 | -17.2145 | 82.6456 | 61.4813 | 41.9982 | -10.8741 | -17.1541 |
| 6 | 42.3499 | -11.5346 | -16.0550 | 83.3189 | 62.8119 | 42.0001 | -11.1952 | -15.9946 |
| 7 | 42.3235 | -11.8403 | -14.8984 | 84.0368 | 64.1201 | 41.9744 | -11.5002 | -14.8381 |
| 8 | 42.2701 | -12.1267 | -13.7478 | 84.7952 | 65.4037 | 41.9216 | -11.7859 | -13.6875 |
| 9 | 42.1887 | -12.3951 | -12.5881 | 85.6028 | 66.6801 | 41.8409 | -12.0536 | -12.5278 |
| 10 | 42.0810 | -12.6382 | -11.4407 | 86.4436 | 67.9268 | 41.7339 | -12.2961 | -11.3803 |
| 11 | 41.9480 | -12.8543 | -10.3082 | 87.3133 | 69.1421 | 41.6015 | -12.5115 | -10.2479 |
| 12 | 41.7909 | -13.0422 | -9.1939 | 88.2076 | 70.3245 | 41.4452 | -12.6986 | -9.1336 |
| 13 | 41.6089 | -13.2026 | -8.0874 | 89.1330 | 71.4859 | 41.2638 | -12.8583 | -8.0271 |
| 14 | 41.4049 | -13.3328 | -7.0046 | 90.0753 | 72.6113 | 41.0606 | -12.9877 | -6.9443 |
| 15 | 41.1804 | -13.4330 | -5.9477 | 91.0304 | 73.6997 | 40.8369 | -13.0872 | -5.8874 |
| 16 | 40.9370 | -13.5036 | -4.9185 | 91.9947 | 74.7505 | 40.5942 | -13.1570 | -4.8583 |
| 17 | 40.6739 | -13.5458 | -3.9114 | 92.9725 | 75.7713 | 40.3320 | -13.1984 | -3.8511 |
| 18 | 40.3945 | -13.5599 | -2.9357 | 93.9529 | 76.7531 | 40.0534 | -13.2118 | -2.8755 |
| 19 | 40.1003 | -13.5476 | -1.9929 | 94.9331 | 77.6961 | 39.7600 | -13.1987 | -1.9326 |
| 20 | 39.7927 | -13.5103 | -1.0838 | 95.9105 | 78.6002 | 39.4531 | -13.1606 | -1.0235 |
| 21 | 39.1392 | -13.3672 | 0.6385 | 97.8564 | 80.3003 | 38.8013 | -13.0159 | 0.6989 |
| 22 | 38.4469 | -13.1456 | 2.2199 | 99.7657 | 81.8471 | 38.1107 | -12.7927 | 2.2802 |
| 23 | 37.7230 | -12.8603 | 3.6640 | 101.6304 | 83.2472 | 37.3886 | -12.5056 | 3.7243 |
| 24 | 36.9768 | -12.5262 | 4.9709 | 103.4379 | 84.5020 | 36.6442 | -12.1700 | 5.0312 |
| 25 | 36.2147 | -12.1572 | 6.1462 | 105.1827 | 85.6166 | 35.8839 | -11.7993 | 6.2065 |
| 26 | 35.4425 | -11.7653 | 7.1962 | 106.8605 | 86.5960 | 35.1135 | -11.4058 | 7.2566 |
| 27 | 34.6657 | -11.3619 | 8.1278 | 108.4676 | 87.4441 | 34.3384 | -11.0005 | 8.1882 |
| 28 | 33.8872 | -10.9554 | 8.9498 | 110.0049 | 88.1668 | 33.5618 | -10.5925 | 9.0101 |
| 29 | 33.1117 | -10.5544 | 9.6688 | 111.4698 | 88.7674 | 32.7881 | -10.1898 | 9.7292 |
| 30 | 32.3404 | -10.1644 | 10.2943 | 112.8656 | 89.2511 | 32.0187 | -9.7983 | 10.3546 |
| 31 | 31.5770 | -9.7917 | 10.8322 | 114.1905 | 89.6205 | 31.2571 | -9.4239 | 10.8925 |
| 32 | 30.8217 | -9.4395 | 11.2912 | 115.4484 | 89.8798 | 30.5035 | -9.0701 | 11.3515 |
| 33 | 29.3430 | -8.8113 | 11.9957 | 117.7634 | 90.0766 | 29.0285 | -8.4389 | 12.0561 |
| 34 | 27.9116 | -8.2994 | 12.4540 | 119.8200 | 89.8573 | 27.6005 | -7.9241 | 12.5144 |
| 35 | 26.5305 | -7.9164 | 12.7036 | 121.6231 | 89.2298 | 26.2227 | -7.5384 | 12.7639 |
| 36 | 25.8590 | -7.7758 | 12.7593 | 122.4293 | 88.7635 | 25.5529 | -7.3966 | 12.8197 |
| 37 | 25.1998 | -7.6704 | 12.7731 | 123.1710 | 88.1947 | 24.8951 | -7.2898 | 12.8335 |
| 38 | 24.5526 | -7.6006 | 12.7474 | 123.8462 | 87.5227 | 24.2494 | -7.2189 | 12.8077 |
| 39 | 23.9167 | -7.5672 | 12.6841 | 124.4524 | 86.7463 | 23.6150 | -7.1844 | 12.7444 |
| 40 | 23.2914 | -7.5707 | 12.5847 | 124.9863 | 85.8639 | 22.9910 | -7.1867 | 12.6450 |
| 41 | 22.6759 | -7.6114 | 12.4504 | 125.4431 | 84.8743 | 22.3768 | -7.2266 | 12.5107 |
| 42 | 22.0685 | -7.6900 | 12.2818 | 125.8175 | 83.7751 | 21.7707 | -7.3042 | 12.3421 |
| 43 | 21.4685 | -7.8067 | 12.0793 | 126.1024 | 82.5655 | 21.1717 | -7.4200 | 12.1396 |
| 44 | 20.8737 | -7.9617 | 11.8425 | 126.2893 | 81.2431 | 20.5779 | -7.5743 | 11.9028 |
| 45 | 20.2826 | -8.1550 | 11.5708 | 126.3676 | 79.8075 | 19.9878 | -7.7669 | 11.6310 |
| 46 | 19.6931 | -8.3865 | 11.2627 | 126.3251 | 78.2573 | 19.3990 | -7.9979 | 11.3230 |
| 47 | 19.1029 | -8.6557 | 10.9165 | 126.1469 | 76.5929 | 18.8094 | -8.2665 | 10.9768 |
| 48 | 18.5047 | -8.9436 | 10.5536 | 125.8207 | 74.9053 | 18.2115 | -8.5541 | 10.6138 |
| 49 | 17.8916 | -9.2238 | 10.2051 | 125.3498 | 73.3208 | 17.5986 | -8.8343 | 10.2654 |
| 50 | 17.2661 | -9.4944 | 9.8697 | 124.7401 | 71.8421 | 16.9728 | -9.1050 | 9.9300 |
| 51 | 16.6296 | -9.7549 | 9.5450 | 123.9959 | 70.4676 | 16.3357 | -9.3660 | 9.6053 |
| 52 | 15.9844 | -10.0048 | 9.2294 | 123.1227 | 69.1979 | 15.6897 | -9.6165 | 9.2897 |
| 53 | 15.3311 | -10.2447 | 8.9205 | 122.1242 | 68.0297 | 15.0353 | -9.8573 | 8.9808 |
| 54 | 14.6718 | -10.4746 | 8.6170 | 121.0068 | 66.9625 | 14.3746 | -10.0882 | 8.6773 |
| 55 | 14.0064 | -10.6954 | 8.3168 | 119.7741 | 65.9923 | 13.7076 | -10.3103 | 8.3771 |
| 56 | 13.3367 | -10.9075 | 8.0190 | 118.4339 | 65.1179 | 13.0362 | -10.5237 | 8.0793 |
| 57 | 12.6624 | -11.1116 | 7.7221 | 116.9914 | 64.3354 | 12.3600 | -10.7294 | 7.7824 |
| 58 | 11.9849 | -11.3080 | 7.4258 | 115.4566 | 63.6432 | 11.6803 | -10.9275 | 7.4862 |
| 59 | 11.3035 | -11.4972 | 7.1294 | 113.8367 | 63.0381 | 10.9966 | -11.1185 | 7.1898 |
| 60 | 10.6193 | -11.6792 | 6.8335 | 112.1445 | 62.5185 | 10.3100 | -11.3024 | 6.8937 |
| 61 | 9.9317 | -11.8540 | 6.5380 | 110.3900 | 62.0821 | 9.6200 | -11.4794 | 6.5983 |
| 62 | 9.2413 | -12.0215 | 6.2445 | 108.5886 | 61.7279 | 8.9269 | -11.6490 | 6.3048 |
| 63 | 8.5474 | -12.1816 | 5.9538 | 106.7534 | 61.4544 | 8.2304 | -11.8114 | 6.0142 |
| 64 | 7.1497 | -12.4789 | 5.3891 | 103.0483 | 61.1483 | 6.8274 | -12.1132 | 5.4494 |
| 65 | 5.7367 | -12.7441 | 4.8609 | 99.4106 | 61.1636 | 5.4091 | -12.3832 | 4.9211 |
| 66 | 4.3056 | -12.9766 | 4.3890 | 95.9838 | 61.5065 | 3.9730 | -12.6201 | 4.4492 |
| 67 | 2.8526 | -13.1754 | 3.9939 | 92.9144 | 62.1887 | 2.5157 | -12.8230 | 4.0543 |
| 68 | 2.1161 | -13.2617 | 3.8317 | 91.5591 | 62.6622 | 1.7774 | -12.9112 | 3.8920 |
| 69 | 1.3736 | -13.3388 | 3.6966 | 90.3502 | 63.2263 | 1.0331 | -12.9899 | 3.7569 |
| 70 | 0.6224 | -13.4061 | 3.5907 | 89.3036 | 63.8850 | 0.2806 | -13.0586 | 3.6511 |
| 71 | -0.1357 | -13.4628 | 3.5168 | 88.4409 | 64.6389 | -0.4788 | -13.1166 | 3.5771 |
| 72 | -0.9037 | -13.5078 | 3.4766 | 87.7786 | 65.4929 | -1.2478 | -13.1625 | 3.5369 |
| 73 | -1.6793 | -13.5396 | 3.4724 | 87.3376 | 66.4465 | -2.0240 | -13.1950 | 3.5328 |
| 74 | -2.4656 | -13.5567 | 3.5059 | 87.1349 | 67.5049 | -2.8107 | -13.2125 | 3.5662 |
| 75 | -3.2596 | -13.5570 | 3.5781 | 87.1889 | 68.6651 | -3.6049 | -13.2130 | 3.6385 |
| 76 | -4.0643 | -13.5384 | 3.6902 | 87.5162 | 69.9313 | -4.4095 | -13.1943 | 3.7504 |
| 77 | -4.8760 | -13.4989 | 3.8414 | 88.1294 | 71.2969 | -5.2208 | -13.1545 | 3.9017 |
| 78 | -5.6972 | -13.4361 | 4.0315 | 89.0416 | 72.7636 | -6.0415 | -13.0910 | 4.0919 |
| 79 | -6.5234 | -13.3484 | 4.2578 | 90.2546 | 74.3195 | -6.8668 | -13.0025 | 4.3181 |
| 80 | -7.3563 | -13.2344 | 4.5179 | 91.7729 | 75.9624 | -7.6988 | -12.8875 | 4.5782 |
| 81 | -8.1908 | -13.0940 | 4.8060 | 93.5819 | 77.6744 | -8.5322 | -12.7460 | 4.8663 |
| 82 | -9.0282 | -12.9276 | 5.1180 | 95.6716 | 79.4488 | -9.3682 | -12.5783 | 5.1783 |
| 83 | -9.4462 | -12.8354 | 5.2803 | 96.8108 | 80.3519 | -9.7855 | -12.4856 | 5.3406 |
| 84 | -9.6506 | -12.7941 | 5.3542 | 97.3130 | 80.7437 | -9.9639 | -12.4445 | 5.4099 |
| 85 | -9.8303 | -12.7930 | 5.3779 | 97.2930 | 80.7648 | -9.9653 | -12.4468 | 5.4019 |
